# Cryogenic Materials for Use in High-Radiation and Low-Magnetic-Field Environments

**DOI:** 10.3390/ma19163422

**Published:** 2026-08-12

**Authors:** Ekaterina Korobkina, Austin Reid, Clark Hickman, Markus Tam, Shane Golio, Cole Teander, Paul Huffman, Thomas Rao, Tushar Mahale, Robert Golub

**Affiliations:** 1 Department of Nuclear Engineering, North Carolina State University, Raleigh, NC 27695, USA; sngolio@ncsu.edu; 2 Department of Physics, North Carolina State University, Raleigh, NC 27695, USA; areid@tntech.edu (A.R.); cteander@triumf.ca (C.T.); paul_huffman@ncsu.edu (P.H.);; 3 Department of Industrial Engineering, North Carolina State University, Raleigh, NC 27695, USA

**Keywords:** cryogenic, metamaterial, origami, non-magnetic material, bellows, superconducting joint, explosively bonded, Ti6Al4V, polyimide, Zircalloy-4, niobium

## Abstract

Ultra-cold neutrons (UCNs) play an important role in the modern frontier of low-energy physics related to fundamental symmetries. They have enabled an improvement of two orders of magnitude in the measurement of the upper limit of the neutron electric dipole moment (nEDM) compared to beam experiments. Further improvements in both the design of UCN sources and nEDM measurements critically depend on the availability and development of materials which satisfy specific requirements. We present innovative materials used for the fabrication of the cryogenic UCN source at the PULSTAR reactor (NC State University, USA) and for the cryogenic non-magnetic environment required for a new high-precision nEDM experiment seeking a further two-orders-of-magnitude improvement in nEDM sensitivity.

## 1. Introduction

Neutrons are widely used for applied, condensed matter, and fundamental physics studies. Their area of application depends on their energy, with the lowest energy neutrons primarily utilized for studies of the fundamental properties of nature. A unique property of these ultra-cold neutrons (UCNs) is that their kinetic energy is in the same range as the so-called nuclear Fermi potential of materials (typically −10 to 350 neV), similar in scale to gravitational (1 cm ≈ 1 neV) and magnetic (1 T = 60 neV) potential energies. Therefore, unlike other neutrons, UCNs can be confined in any combination of the potentials mentioned above for long periods of time. Observing them under these conditions allows certain fundamental constants of nature to be studied with very high precision. While at present UCNs are used mainly for fundamental physics studies, there is also potential to use them for condensed matter studies, although such instruments typically require a higher intensity of UCNs than is available at existing sources [1,2]. To achieve greater densities of UCNs, modern sources utilize superthermal production [3] which requires a dedicated cryogenic facility and either deuterium cooled to a temperature of ∼5 K [4,5] or liquid helium (LHe) below 1 K [6,7].

Both the cryogenic nature of the source and unique materials constraints set by the experiments themselves present significant engineering challenges. For example, a typical UCN source requires materials that have high radiation resistance, low neutron activation levels, and low neutron absorption, and function well at cryogenic temperatures. Precision neutron experiments can add additional or higher-level constraints such as non-metallic regions to minimize eddy currents, material choices that have zero activation, or the need to move and change shape while at ultra-low temperatures. Such combinations greatly reduce the choice of materials available, resulting in the inability to utilize standard cryogenic techniques that rely on metals such as stainless steel (too high neutron activation and neutron absorption) or plastics/epoxies (not radiation-resistant, too high neutron absorption).

One of the frontier experiments utilizing UCNs is the study of the neutron electric dipole moment (nEDM). This experiment probes for an asymmetry in the neutron that would arise if the fundamental CP (Charge-conjugation/Parity) symmetry is violated. Such a violation could, for example, explain the matter/anti-matter asymmetry in our universe. As part of our effort to develop an apparatus that would allow an nEDM measurement with a sensitivity improvement of two order of magnitude [8] compared to the existing best result [9], we developed the four unique cryogenic components that are presented in this work.

For the new cryogenic nEDM experiment [10], the most challenging requirement is that the cryogenic apparatus must have a significant liquid-helium-filled volume (∼1000 L) with an extremely stable, homogeneous, and low-magnitude (1 μT) permanent magnetic field, $B_{0}$, as well as a precise oscillating RF field for spin manipulation. The required $B_{0}$ field stability is achieved through the use of a superconducting shield and a superconducting $B_{0}$ coil operated in persistent current mode [11]. The presence of the RF field requires that no metal be used around the experimental volume and that all components must be RF-transparent, including the flexible actuation devices. To verify the feasibility of this new nEDM technique, our team has developed a cryogenic test bed with superconducting $B_{0}$ and RF coils [12].

Our cryogenic test bed for the nEDM experiment—currently located at at the Triangle University Nuclear Lab (TUNL) (Section 3)—will be coupled to the UCN source at NC State University’s PULSTAR reactor (Section 2). Radiation-resistant cryogenic metamaterial supports for the horizontal section of the UCN source cryostat are described in Section 2.2, and a temperature-gradient bridge between 5 K and 40 K parts of the cryostat is described in Section 2.3. Material requirements for environments that are extra sensitive to magnetic disturbances such as those used in nEDM experiments are described in Section 3.1. A flexible, plastic metamaterial to be used as a non-metallic bellows is described in Section 3.2. A novel fabrication technique to create superconducting joints for easy reassembly of a low-current persistent current switch is described in Section 3.3.

## 2. Cryogenic Materials Development for the UCN Source at the PULSTAR Reactor

While individual UCN sources must be uniquely designed to match their respective facility, there are universal material and design considerations inherent to all UCN sources. Some of these UCN-based considerations (described in Section 2.1) led the UCN source at the PULSTAR reactor to be designed with an elbow-shaped cryostat [13,14]. This shape presented unique cryogenic and mechanical challenges that motivated the development of a novel Ti6Al4V (Ti with 6% Wt Al and 4% Wt V) metamaterial structure. The design and subsequent testing of this structure is described in Section 2.2. Similarly, the need to thermally isolate the bottom (at 5 K) and top (30–50 K) of our elbow led us to investigate the application of Zircaloy-4 explosively bonded to Al6061, the results of which are described in Section 2.3.

### 2.1. UCN Source Material and Design Considerations for the PULSTAR Reactor

To produce UCNs, a stream of thermal neutrons must irradiate the UCN source cryostat. Within the cryostat, cryogenically thermalized cold neutrons (1–10 meV range) are converted to ultra-cold neutrons with energies less than a few hundred neV by superthermally downscattering on lattice excitations [3,15,16]. Only materials with zero or negligible neutron cross-sections can be used for UCN conversion. Nature has not provided us with many suitable options: in practice, the only suitable converters are either liquid helium (LHe) [15] or solid deuterium (sD_2_) [17]. When UCNs leave a converter, they receive a boost in kinetic energy equal to the Fermi potential of the converter material. For helium, this is about 15 neV; for deuterium, it is significantly higher at 110 neV. UCNs with kinetic energies below the Fermi pseudo-potential of their containing material can be fully reflected under any angle of incidence, so UCNs can be delivered to an experimental area by highly polished tubes made or coated with materials with a high neutron Fermi pseudo-potential. The best guide or coating materials are Ni-58 ($E_{F}=350$ neV), Be ($E_{F}=250$ neV), and Nickel alloys such as NiMo and NiP ($E_{F}=220$ neV). Because UCNs leaving an sD_2_ source receive a kinetic energy boost of 110 neV, the choice of guide material and geometry is especially important. For example, an sD_2_ source leading horizontally into a Ni-58 guide can extract UCNs only in the range of 110 to 350 neV. The best strategy for maximizing UCN yield, then, is to use the gravitational potential of UCNs to compensate for the kinetic energy boost by first extracting UCNs vertically before transporting them in horizontal guides.

This vertical-to-horizontal transition can be limited in that a UCN source must fit into the already-existing neutron beam structures at its reactor. For example, at the Mainz University TRIGA [18] and Munich Technical University FRM-II [19] reactors, UCN source cryostats can only be inserted into horizontal beam tubes with a typical diameter below 15 cm. As a result, the cryostats are constructed with a horizontal cylindrical shape.

Unique to the NC State University PULSTAR reactor, we were able to repurpose the 122 × 122 × 200 cm thermal column cavity for the placement of the UCN source. As a result, it was possible to design the source with the optimum geometry of the vertical-to-horizontal transition described above. The resulting cryostat shape is an elbow, optimized for the transportation of UCNs but complicating the cryogenic design. To produce UCNs, our elbow-shaped cryostat must be cooled to 50 K at the hot end and 5 K at the cold end. The cross-sectional view of the cryostat can be seen in Section 2.2.3 and the detailed design is described in [13].

With design considerations in mind, we needed to create an efficient mechanical support for a thermal gradient between 300 K and 50 K over a length of 4 cm with a heat leak below 1 W. In addition, supports must have an area of several square cm to hold and align the cryostat with a weight of about 15 kg inside of the elbow-shaped vacuum jacket, and allow multiple assemblies and disassemblies during cryogenic commissioning. The design must provide some flexibility to accommodate changes in the outer diameter of the cryostat during cooling. To satisfy such a complex set of requirements, a special thermal–mechanical metamaterial was designed. Several prototypes were built and cryogenically tested for thermal conductivity and flexibility.

### 2.2. Thermal Metamaterial for Cryostat Supports

#### 2.2.1. Design Considerations and Fabrication

Metamaterials are a new class of materials which obtain properties different from the original material due to a special geometry pattern [20]. As a base material, Grade 5 titanium alloy (Ti6Al4V: Ti with 6% Wt Al, 4% Wt V) was chosen due to its high strength-to-weight ratio, low thermal conductivity [21], and low neutron activation [22]. The Ti6Al4V alloy also possesses particularly good irradiation resistance for our operational conditions. Ti6Al4V irradiation damage is caused by high-energy (>0.5 MeV) fast neutrons [23,24], but at our facility such neutrons contribute a maximum of 3% of the total flux depending on the location [14]. It would take 30 years of 24/7 operation of the PULSTAR reactor to reach the irradiation-damage threshold neutron fluence of $10^{17}$ n/cm^2^ measured in [23]. In reality, the PULSTAR reactor is only operated 8 h per day and 5 days a week. Therefore, we do not expect any noticeable radiation damage.

The total heat power, *Q*, flowing through a conductor from the high-temperature end at $T_{2}$ to the low-temperature end at $T_{1}$ can be written as

(1) $$Q=-\frac{A}{L}\int_{T_{1}}^{T_{2}}\kappa \left(T\right)\;dT$$

where κ(T) is the temperature-dependent heat conductivity, *A* is the cross-sectional area, and *L* is the length of the conductor. The heat integral for the Ti6Al4V alloy from $T_{2}=300$ K to $T_{1}=50$ K is in the $10^{3}$ W/m range [25]. To keep the heat leak ≈ 1 W (with safety factor 2), the geometric factor (g=A/L) must be on the order of $10^{-4}$ m; taking into account the required distance of L=0.04 m, the effective thermal cross-sectional area must not exceed $4\times 10^{-6}$ m^2^ (4 mm^2^). To achieve such a low effective thermal cross-section while also satisfying our requirement of several cm^2^ of area for support, metal foam presented itself as an attractive geometric prototype. Compared to solid samples, metal foams can have a significantly smaller density, but they are not flexible.

Another department at NC State University had been developing an additive Electron Beam Manufacturing (EBM) technique for non-stochastic foams, i.e., lattices for building customized medical implants from Grade 5 titanium [26]. The stiffness of such structures can be changed by varying the thickness, length and angles between lattice struts. Mechanical stress tests showed that such structures looked promising for our application [27]. Therefore, a similar non-stochastic hexagonal pattern was used to manufacture a slightly flexible metamaterial with a high thermal resistivity for our cryostat supports.

For our supports, we decided to use a 45-degree build angle to increase compression resistance in the built direction and to make mechanical properties more isotropic. Two strut diameters—0.7 mm (the smallest possible for this EBM machine) and 1 mm—were used for testing. All samples have flat flanges (with mounting holes) on the top and bottom to enforce mechanical stability.

**Figure 1:**
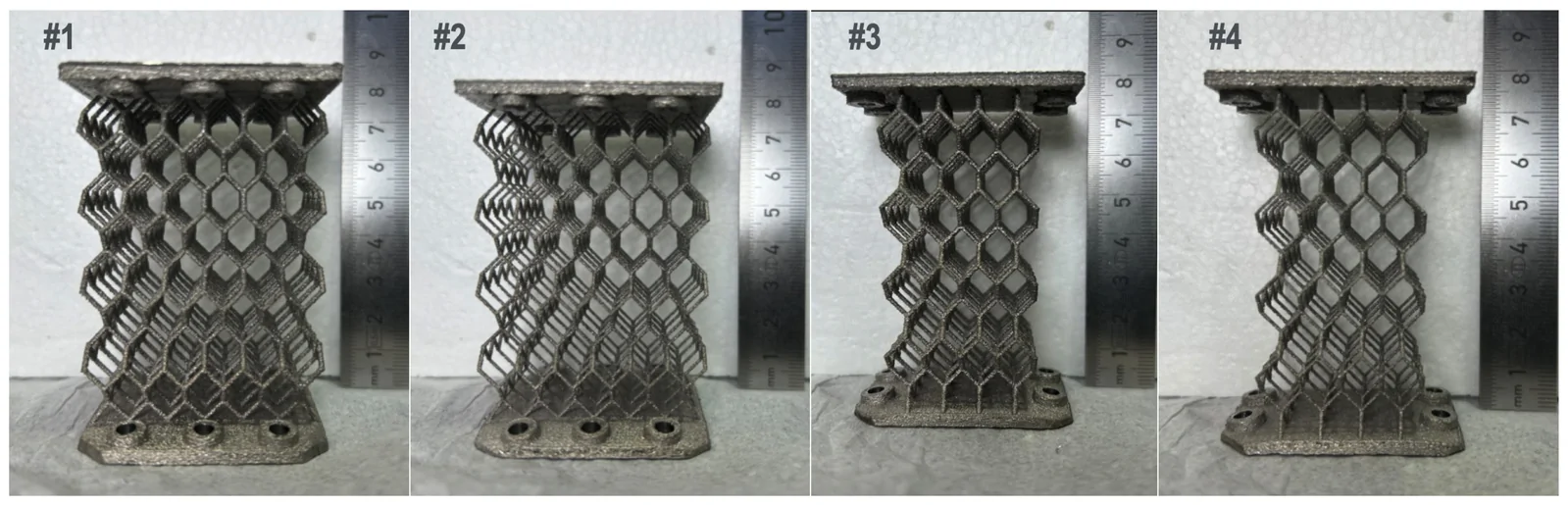
Four thermal metamaterial samples fabricated for *g*-factor testing. (**#1**): A 6 × 4 × 8 lattice with a strut thickness of 1 mm; (**#2**): 6 × 4 × 8, strut 0.7 mm; (**#3**): 4 × 4 × 8, strut 1 mm; (**#4**): 4 × 4 × 8, strut 0.7 mm.

Four samples of two geometries (6 × 4 × 8 and 4 × 4 × 8 lattice dimensions as shown in Figure 1) were designed specifically for measuring the geometric factor, *g*, to validate the theoretical thermal design. One cell is defined as a hexagonal ring of struts, each measuring 7.6 mm wide and 15.2 mm tall. These cells are interlinked to form a three-dimensional scaffold structure. The top and bottom flanges have holes for mounting to the cryogenic setup. The flange dimensions were 5×5 cm for the 6×4 samples and 5×3.5 cm for the 4×4 samples. The samples were built on Arcam AB’s EBM S12 system. The design procedure and building parameters were identical to the ones described in [27].

#### 2.2.2. Method

Ti6Al4V alloy solid samples can become fragile below 20 K [23]. In our UCN source cryostat, the lowest temperature for the supports will be about 50 K. Nevertheless, it was necessary to test if the EBM-built structures could stay flexible at cryogenic temperatures after being compressed at room temperature. To test cryogenic flexibility, a special small press was fabricated from Al6061 alloy, which has a larger thermal contraction than Ti6Al4V. The samples were compressed by 2 mm at room temperature and then dunked into liquid nitrogen. The sample with 0.7 mm struts demonstrated several vertices collapsing, while no damage was observed with the 1 mm structures. While this ultimately led us to choose the 1 mm strut for the real cryostat supports, both thicknesses were used for the testing of the geometric factor.

**Figure 2:**
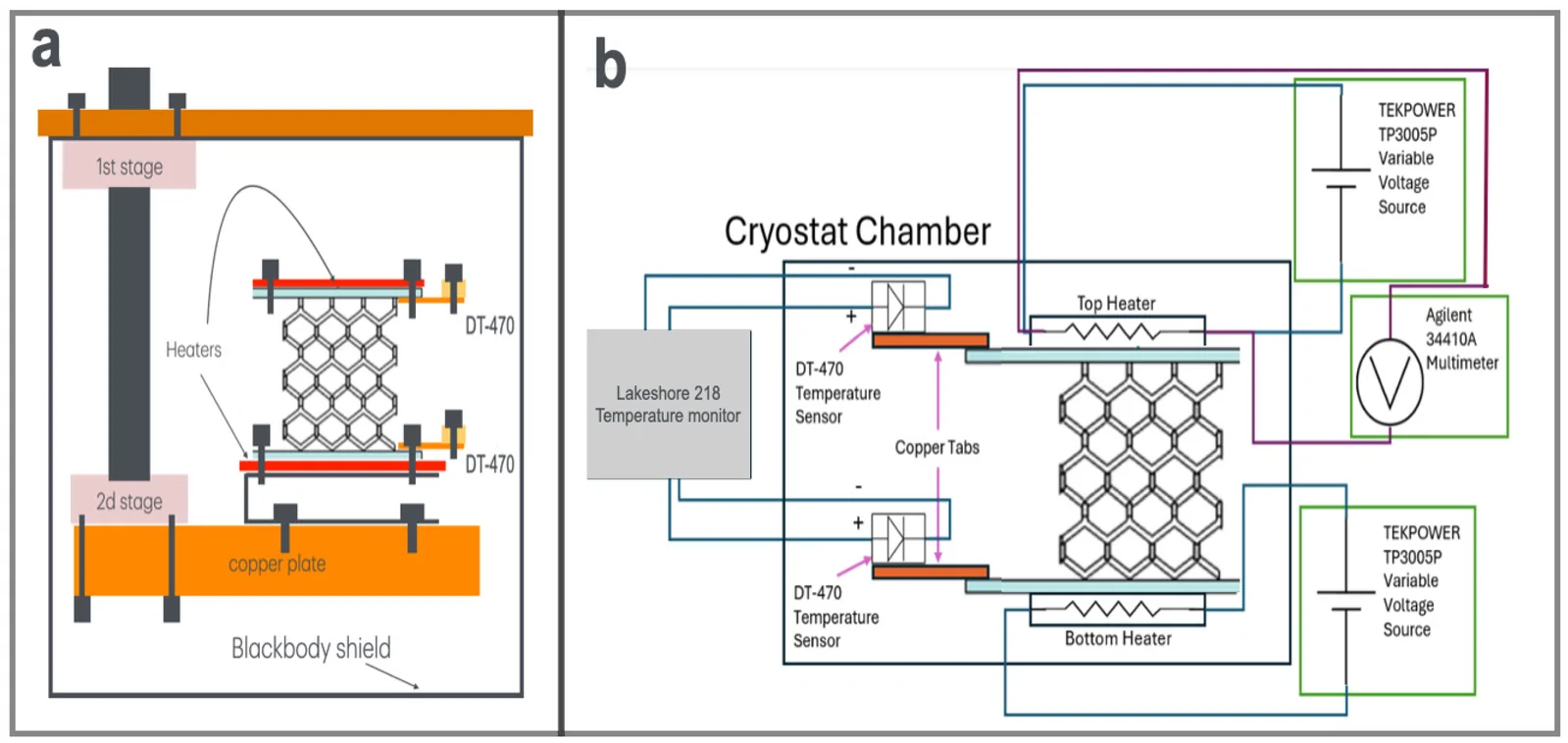
Schematic of the experimental setup to measure *g*-factor of the EBM-built thermal metamaterial. (**a**) Cryogenic and (**b**) electrical setups.

A schematic of the experimental setup to measure the geometric factor of Ti6Al4V structures is shown in Figure 2. We used a two-stage Sumitomo RDK 415D cryocooler (Shinagawa-ku, Japan) for this cryogenic experiment. This modified Gifford–McMahon cryocooler provided up to 45 W of cooling power at 50 K to the first stage and 1.5 W at 4.2 K to the second stage. The second stage was equipped with a thick copper mounting plate that served as a cold-end heat sink. The plate temperature was about 4 K during operation. The first stage was used to cool down a black-body shielding structure that surrounded the sample setup and was kept at about 65 K.

It is known that Ti6Al4V alloy becomes superconducting below 4.36 K [21]. To avoid superconductivity, a stainless steel C-shaped offset was mounted on the copper plate to increase the lowest temperature for the Ti6Al4V alloy. The bottom and top flanges of the sample were equipped with heaters made from NiCr wire pressed into indium and sandwiched between two Cu foils to increase thermal diffusivity. The top heater was electrically mounted using a four-wire setup: two wires were connected to the power supply while other two were connected to a multimeter to measure the voltage across the heater as shown in Figure 2b. Lakeshore DT-470 diodes were mounted on the top and bottom flanges of specimens. All leads were heat-sunk at the first-stage flange of the cryocooler. The cryostat vacuum was kept at $10^{-6}$ mbar during cooldowns.

The samples were cooled until temperatures stabilized; the corresponding temperature values were taken as a zero-power steady-state measurement. Because of their very small *g*-factor, our samples still had gradients of about 30 K due to residual heat leaks in the mW range from the wiring. After establishing the zero-power steady state, three incremental currents were applied to the top heater resulting in three temperature gradients. Each current was applied until the sample reached a new stable temperature.

The incremental measurement of temperature gradients allowed us to derive the $g_{\exp}$ value. The power, *Q*, required to produce the experimental temperature gradients can be written using Equation (1) as

(2) $$Q_{0}=-g_{\exp}\int_{T_{\mathrm{top}}^{0}}^{T_{\mathrm{bottom}}^{0}}\kappa \left(T\right)\;dT,$$

for the first run with no additional power from the heater wires, and as

(3) $$Q_{i}=Q_{0}+P_{i}=-g_{\exp}\int_{T_{\mathrm{top}}^{i}}^{T_{\mathrm{bottom}}^{i}}\kappa \left(T\right)\;dT,$$

for the runs with the applied heater power $P_{i}$. Assuming that $Q_{0}$ is the same for all runs and subtracting data from two runs, we can estimate $g_{\exp}$ as

(4) $$g_{\exp}^{i}=\left(P_{i+1}-P_{i}\right)/\left(\int_{T_{\mathrm{top}}^{i}}^{T_{\mathrm{bottom}}^{i}}\kappa \left(T\right)\;dT-\int_{T_{\mathrm{top}}^{i+1}}^{T_{\mathrm{bottom}}^{i+1}}\kappa \left(T\right)\;dT\right).$$

In the ideal case, all $T_{\mathrm{bottom}}^{i}=T_{\mathrm{bottom}}^{0}$. In reality, a small difference might exist, but this is not expected to have much impact on the final result because temperatures are only an integration limit and κ(T) is a smooth function.

#### 2.2.3. Results and Discussion

All raw data from measurements are shown in Table 1. To calculate $g_{\exp}$, we need to know the values of the thermal conductivity integrals. The typical temperatures of the bottom and top flanges were around 30 K and 65 K. Since Ti6Al4V is not as common of a cryogenic material as stainless steel, only limited thermal conductivity data exists. For the temperature range of interest, the only measurement of thermal conductivity of the common Ti6Al4V alloy is from 1963 [28]. This measurement claims to be accurate in the range of 20 K and 300 K with 5% accuracy. It has several data points at ∼22 K and then two points around 80K. There is also a 1992 measurement [29] where data points were taken using small temperature increments from 3 K up to 65 K. However, this study tested a variation of Grade 5 titanium: the Ti6Al4V ELI alloy, with a reduced amount of impurities of oxygen and iron. The most recent measurement of the room-temperature value was published in 2020 [30]. Since amount of impurities can alter the cryogenic data, we opted for a fit of the 1963 and 2020 data and used the analytical fit function $\kappa \left(T\right)=0.376+0.0524T-1.8534\times 10^{-4}T^{2}+3.050\times 10^{-7}T^{3}$.

Raw data and experimental $g_{exp}$ for four heat flux configurations for all samples are shown in Table 1. Formula (4) uses data from two measurements. The results shown in Table 1 are calculated using following pairs of runs: (1,2), (2,3) and (3,1), where zero index denotes a run with zero heating power. The latter is used only to show the initial conditions with the static heat leaks at zero power and is not used for calculations.

3D models of the Ti6Al4V samples were imported to COMSOL^™^ (version 5.0) software to simulate the heat fluxes producing the temperature gradients and obtain theoretical geometric factors $g_{\mathrm{sim}}$. These values—along with the averaged experimental values and the ratio of the two—are shown in Table 2.

From the experimental data shown in Table 2, we see that our thermal design is consistent with our goal to have heat leak from 300 K to 50 K ≈ 1 W. Indeed, the largest experimental *g*-factor (Sample #1) is 0.5 mm for the 8 cm length. Our supports will have 4 cm length and a *g*-factor of 1 mm. The resulting heat leak of ≈1.3 W does not significantly exceed 1 W and is within our engineering safety factor of 2. In addition, an actual heat leak between 300 K and 50 K surfaces can be reduced further by introducing an additional thermal resistivity at the interfaces between the support and 50 K walls; i.e., the resulting heat flux will depend on how well the supports are thermally linked to the cryostat.

We also see that there is better agreement between $g_{\exp}$ and $g_{\mathrm{sim}}$ for Samples #1 and #3 with nominal 1 mm strut thickness, which agrees within 30% with the simulated *g*-factors. For the nominal 0.7 mm strut thickness agreement is within 60%.

**Figure 3:**
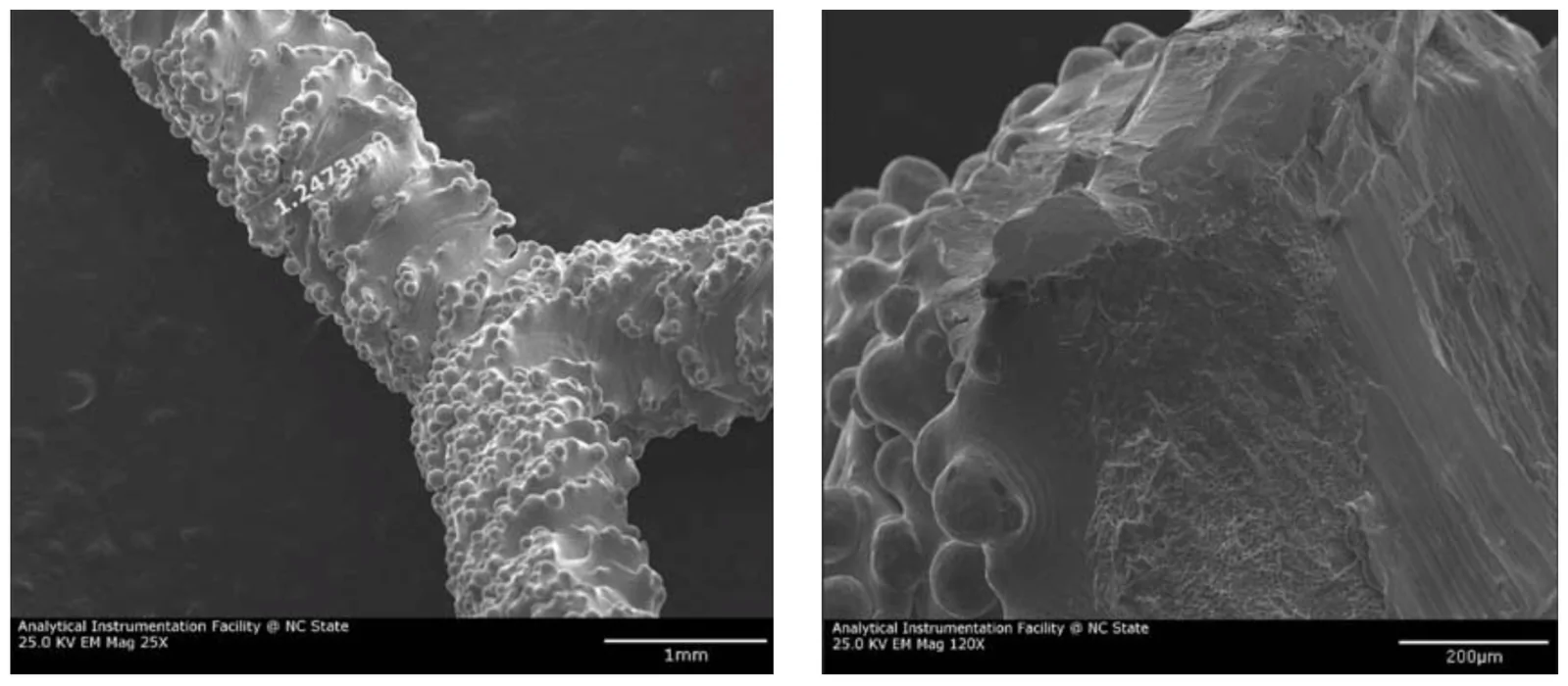
Scanning Electron Micrographs of lattice structure reveal particles sintered to the lattice structures; this increases the effective surface area of the part.

There are several reasons for the possible differences between the simulated and experimental *g*-factors and why they might be more pronounced for the 0.7 mm struts. The biggest effect is expected to be the difference between the nominal strut thickness and that of the built structure. As was pointed out in [27], the fabricated strut thickness depends on the strut angle relative to the horizontal plane. This effect is even more pronounced for the nominal 0.7 mm thickness. In addition, the surface roughness of the struts is significant due to the fabrication process of melting powder particles as shown in Figure 3. This surface roughness introduces uncertainty into the cross-sectional dimension of the final strut. Finally, for Arcam AB’s EBM S12 system, the 0.7 mm electron beam diameter is the minimum fabrication limit, leading to an asymmetrical uncertainty biased towards a physical area larger than the nominal one. These effects generally increase the cross-sectional area and effective $g_{\exp}$-factors of the built struts, particularly for those at 0.7 mm thickness.

Fabrication can also affect the thermal conductivity of the material due to increased porosity and/or the relative area of grain boundaries causing increased scattering of phonons. This would lead to smaller *g*-factors relative to the simulated values. As we did not see an overall decrease in our data, we expect the thermal conductivity difference, if any exists, to be small.

We concluded that there was sufficiently good (for engineering purposes) agreement with the theoretical predictions for struts with thickness above the limiting 0.7 mm. This validates the use of 3D models for the mechanical and thermal design of hexagonal lattice metamaterials with required *g*-factors.

**Figure 4:**
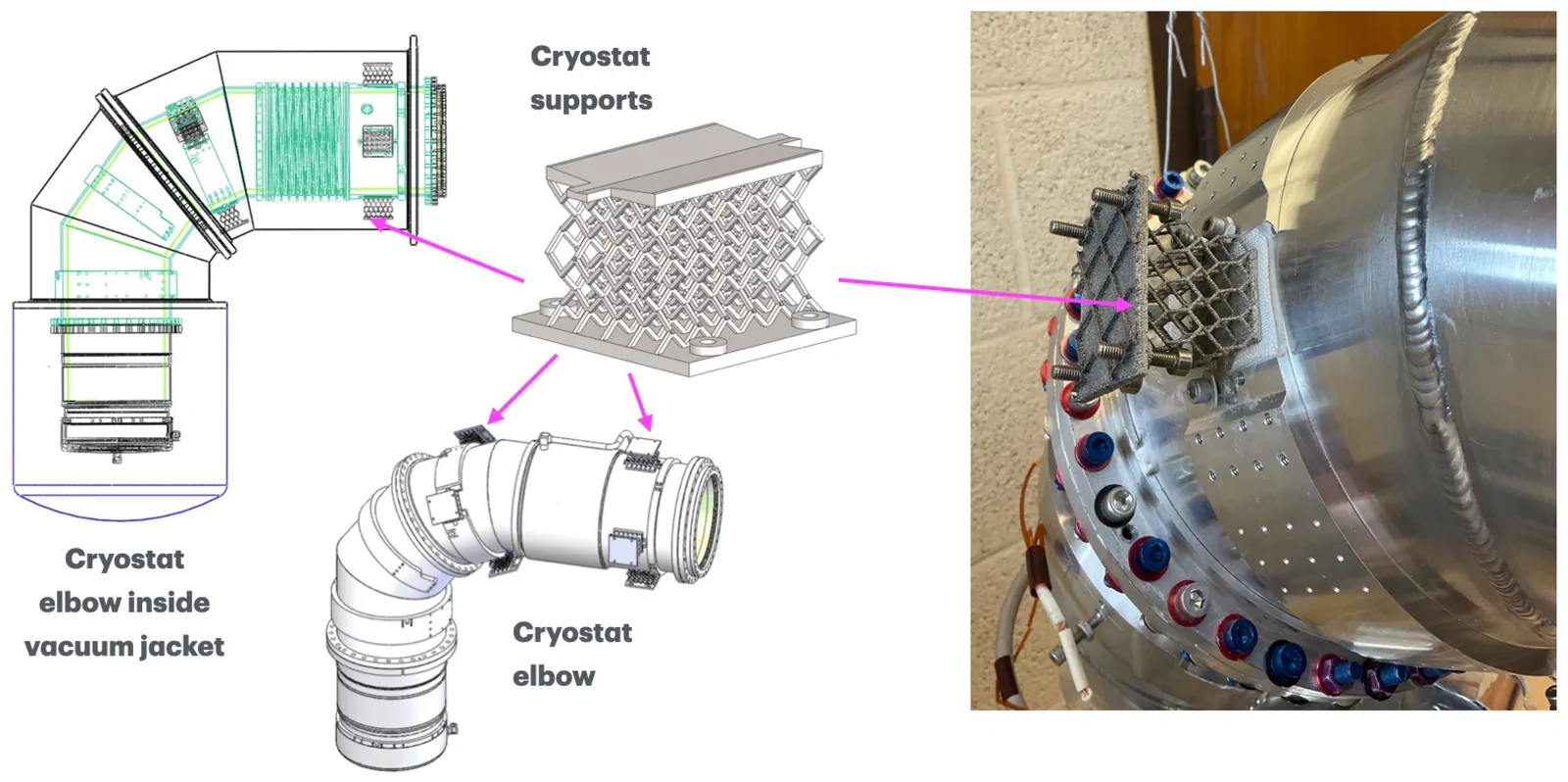
Supports fabricated for UCN source cryostat. (**Left**): 3D rendering of the cryostat elbow assembled with supports inside the vacuum jacket. (**Center top**): 3D rendering of support lattice (see text for dimensions). (**Center bottom**): 3D rendering of cryostat elbow with supports. (**Right**): Supports attached to the cryostat elbow.

Based on our results, the real supports for the cryostat were designed and built as shown in Figure 4. Since the 0.7 mm thick struts demonstrated poor results in the flexibility test and more uncertainty between the experimental and simulated *g*-factor results, we opted to build supports with a strut thickness of 1 mm and 4 × 6 × 4 cells. The bottom flange has tapped holes to add titanium fasteners, which allow spacing adjustment for support between the cryostat and the vacuum jacket. The top flange has features to allow the support to be attached to the cryostat as shown in Figure 4.

### 2.3. Cryogenic Application of Zircaloy-4 Explosively Bonded to Al6061

#### 2.3.1. Material Consideration for Thermal Dridge

In cryogenics, two classes of materials are widely used in the fabrication of experimental setups: those with very high and those with very low thermal conductivities. High-thermal-conductivity materials are required for keeping components thermally linked, while low-thermal-conductivity materials are used as thermal insulators to minimize heat exchange between warmer and colder parts. As described in [13], the bottom 5 cm of the UCN source cryostat is designed to be kept at 5 K during operation, but the rest of the elbow must be kept at temperatures above 30 K. We used an Al6061 alloy as a high-conductivity material for both the 5 K and 30–50 K pieces of the elbow. Al6061 is often a material of choice for reactor applications due to its combination of relatively high thermal conductivity, ductility, low neutron absorption, and the absence of long-lived neutron activation products.

We needed a material with low thermal conductivity to minimize the heat leak from the 30 K and 5 K aluminum sections of the cryostat. Traditionally, either plastic or stainless steel is used for maintaining sufficient cryogenic gradients with minimum heat flows. Plastics are not suitable for irradiation, and stainless steel’s activation and high neutron absorption cross-section are an issue. The Ti6Al4V alloy is suitable due to its activation and thermal properties, but it has high neutron absorption. While this is not an issue in the spacer locations described in Section 2.2, we cannot use Ti6Al4V in close proximity to the 5 K chamber as the material would absorb a significant part of the thermal flux and reduce UCN yield.

Zircaloy-4 (Zry-4) is widely used in the reactor design of fuel elements due to its low neutron absorption and good mechanical strength [31]. Properties of Zry-4 are usually investigated for operational conditions of the nuclear industry, i.e., at elevated temperatures [32]. Studies of cryogenic applications of Zry-4 are rare. Recently, cryogenic temperatures of liquid nitrogen were used for plate rolling as a possible way to refine alloy microstructure [33,34]. Results for Zry-4 cryogenic impact factor tests between 17 and 23 K are described in [35], which included the effect of irradiation fluence up to 10^22^ n/cm^2^ to study the possibility of using Zry-4 for hot and cold neutron sources at the FRM-II research reactor. The impact factor was tested on a plate of 10 mm thickness, and the fracture toughness was estimated to be within required values for cold source operational conditions. The thermal conductivity of Zry-4 is about 10 times less than that of Al6061 at 300 K.

Due to this combination of good neutronic and thermal properties, Zry-4 was considered to be a good candidate for thermal gradient ring fabrication. Nevertheless, there were still two issues to be resolved to meet the required parameters of our UCN source.

The main fabrication problem was the question of how to join these two dissimilar materials to be vacuum-tight (a leak rate below 10^−8^ mbar L/s). Even Al6061 vacuum-tight welding is not easy. We could not find any references to commonly used methods of dissimilar material welding for our combination of aluminum and zirconium alloys. The main issue arises from the large difference in two feedstock materials’ chemical and thermo-physical properties, including melting temperatures, thermal conductivity, and coefficients of thermal expansion (CTE). The latter was also the cause for the second issue: the CTE of zirconium is four times less than that of aluminum [36], which introduces significant strain and shear stresses at the bond interface. This can cause cracking during cooldown. These cracks can either remain detectable at room temperature after warming up or become “cold leaks,” which only open after cooling down below 70 K, making them difficult to identify.

#### 2.3.2. Methods

**Figure 5:**
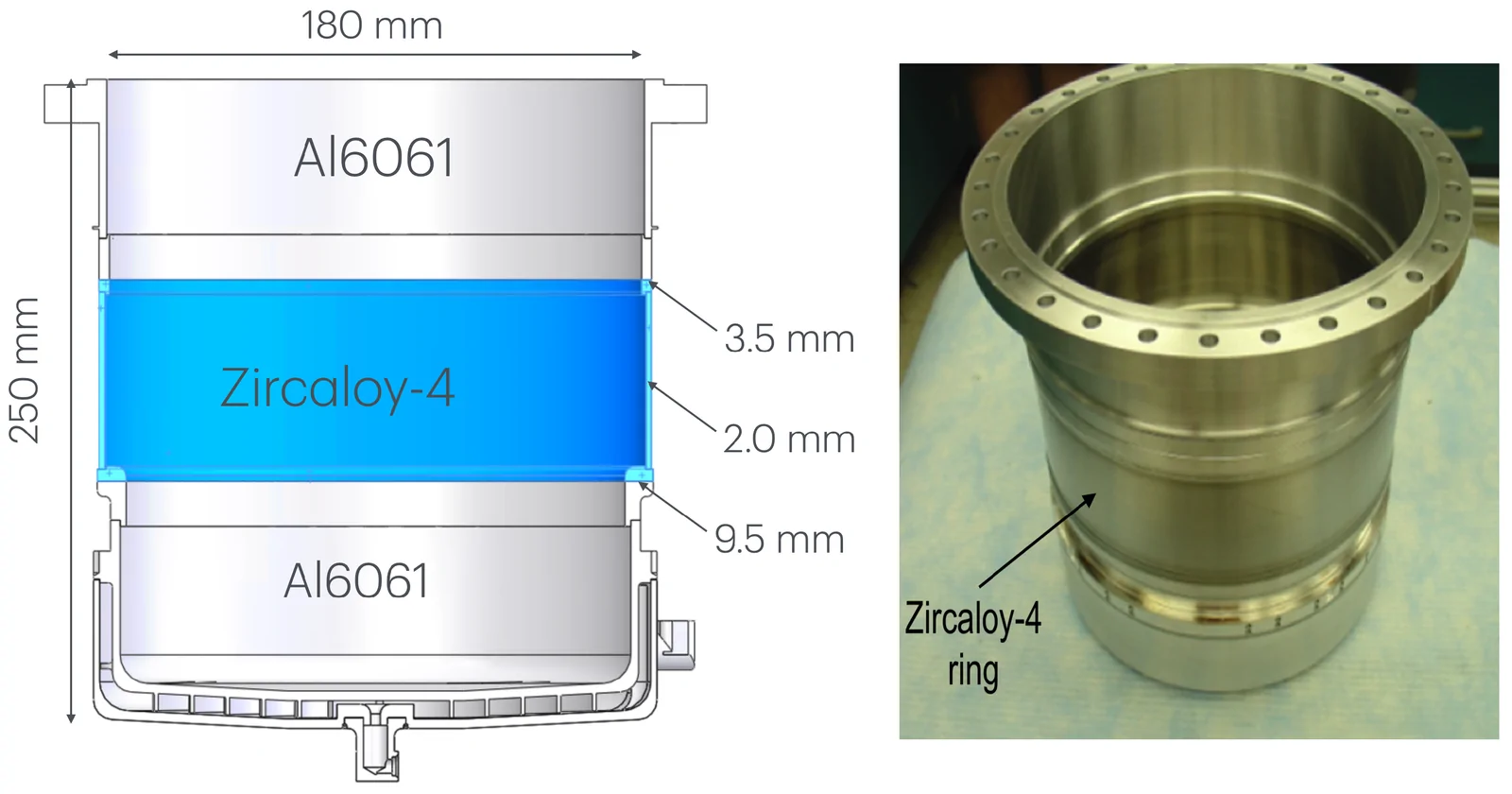
Zircaloy-4 temperature gradient ring EB-welded to the Al6061 cylindrical walls above and below. (**Left**) 3D rendering cross-section with dimensions, where 3.5 and 9.5 mm are the thicknesses of the Zry-4-to-Al top and bottom bonds and 2 mm is the thickness of the Zry-4 ring. (**Right**) Fabricated part.

We found that an explosive welding technique is commonly used for joining thick plates to aluminum alloys [37]. Interestingly, in most cases, the interface of such joints is stronger than the parent metal and cladding due to strain hardening. We found a vendor to try explosive bonding of a 6 mm thick Zry-4 plate to a 25 mm thick Al6061 plate. A thin Ti interlayer was added between the two plates to improve adhesion. The resulting plate was scanned ultrasonically to find two sufficiently large areas with no cracks, where we cut 3.5 mm and 9.5 mm thick rings of ≈18 cm for our cryostat (see Figure 5).

The rings were electron-beam (EB)-welded first to a 55 mm long Zry-4 ring and then to the cylindrical Al6061 walls of the cryostat. The fabricated part is shown in Figure 5. EB welding was used to avoid deformation of the welded parts as well as to ensure vacuum tightness. Mass spectrometer leak checking at room temperature proved the absence of leaks in the 10^−8^ mbar L/s range.

#### 2.3.3. Results

The cryogenic testing of this explosively bonded joint was done by slowly cooling the vacuum-sealed cryostat component in liquid nitrogen vapor and then dipping the bottom (which will be at 5 K in the UCN source) into the liquid. The liquid nitrogen was contained within a polyethylene-lined styrofoam box with a sealed hole to allow for a He gas line during leak checking. A small leak of 10^−7^ mbar L/s was detected during the initial cooldown at the top and more narrow Al-Zry bond. After the leak location was identified, it was patched with a small piece of indium and an aluminum (Al1100 alloy) clamp. The leak likely stemmed from a micro-defect not detected by the ultrasonic scanning. During subsequent cryogenic cycles for solid deuterium studies [38], no leaks were detected above the 10^−9^ mbar L/s range. Therefore, we report the first successful (vacuum-tight) use of Zircaloy-4 explosively bonded to Al6061 at cryogenic temperatures down to 5 K.

## 3. Cryogenic Materials for Low-Magnetic-Field Applications

One of the main requirements for a successful nEDM measurement is the stability and uniformity of the magnetic field. In 1994, a new proposal for an nEDM measurement technique utilizing a unique cryogenic approach was published [8]. The technical realization started later with the formation of the nEDM@SNS collaboration; details of the nEDM@SNS apparatus can be found in [10]. A smaller apparatus (referred to in this paper as the nEDM test bed) with a much quicker operational cycle was designed and built to complete systematic and operational studies before and during the operation of the nEDM@SNS apparatus [12]. The purpose of this device also included the testing of novel techniques and structures—such as the origami bellows and superconducting joints described in this section—that may be utilized later for nEDM@SNS. One of the main advantages of the cryogenic proposal was the possibility of using superconducting materials to achieve temporal stability of the magnetic fields.

### 3.1. nEDM Requirements

The main part of the nEDM@SNS apparatus is the ∼1000 L LHe vessel surrounded by the superconducting magnetic shield and coils. One coil, referred to as the $B_{0}$ coil, provides a stable and homogeneous magnetic field of $10^{-6}$ T. The best magnetic field stability is achieved by operating $B_{0}$ in persistent current mode, where the superconducting coil is electrically disconnected from the power supply after the appropriate current is achieved. Another coil, $B_{\mathrm{RF}}$, generates an oscillating radiofrequency (RF) magnetic field for spin manipulation. Additionally, the apparatus is enclosed in the earth-field cancellation coils and a magnetic shielding room.

The presence of the RF field and the uniformity requirements exclude the use of metal parts inside the 10^−6^ T protected volume. Nevertheless, there are valves that need to be actuated. The conventional solution of beryllium copper bellows is unacceptable due to the field distortions and parasitic heating a conductor would introduce to the fiducial volume. We developed prototypes of the valves for the nEDM@SNS test bed [12]. We again utilized a special metamaterial for this purpose, but instead of the lattice structure we used an origami-folding fabrication technique (see Section 3.2).

Running coils in persistent current mode requires a special superconducting (SC) device called a heat switch, which allows one to connect and disconnect an SC coil from the current source without losing the electric current. It operates by manipulating a section of the coil to be in a superconducting state (the switch is “closed”) or normal (“open”) one [39]. During the commissioning phase of an experiment, there are always situations which require us to connect and disconnect the switch from the SC coils. As detailed in Section 3.3, the existing methods of handling SC joints are not easy to employ in the nEDM test bed, and we will need to disassemble the coil from the heat switch during commissioning. These methods also operate at different current ranges than our experiment. Therefore, we developed a unique method of fabricating SC joints that can be assembled and disassembled more easily and remain suitable for our high-temporal-stability and low-current (<1 A) experiment.

### 3.2. RF-Transparent Cryogenic Origami Metamaterial

#### 3.2.1. Design Requirements for Cryogenic Non-Magnetic Bellows

Needing a flexible non-metallic and non-magnetic device in a superfluid helium bath, we have developed a new class of cryogenic actuators using an origami-folded metamaterial made of Kapton film (Kapton is DuPont’s trade name for a thin-film polyamide preparation). A theory of mechanical stability of origami-folded bellows was developed in [40]. We applied that theory to develop a cryogenic origami metameterial device destined for use in LHe. Because these terms are not commonly used in cryogenics, some clarification is warranted: in its original sense, “origami” means “folded paper,” but in contemporary materials research, the meaning is expanded to admit any substrate that can be folded while still banning topology-changing operations like cutting or gluing. While mechanical properties are traditionally determined by the substrate material, mechanical metamaterials are objects with mechanical properties that have been tuned by the application of a regular deformation. Although it is not as resilient as a conventional beryllium copper device, this folded bellows is well suited to precision experiments as it is both RF-transparent and non-magnetic.

During the warm-up phase of a typical experimental cycle, the bellows needs to be able to withstand elevated internal pressure as the liquid expands and boils. The vacuum system is designed to regularly operate with a pressure differential of 1 bar, so the bellows needs to withstand at least 2 bar without rupturing. Measured perpendicularly to the film, Kapton has a measurable (read: finite) elastic modulus even at 4.2 K [41]. The film’s flexibility at such low temperatures is what makes this bellows’ mechanism of action possible.

Other folded polymeric bellows use edge-glued sheets and sharp folds, but both readily fail during cryogenic actuation. Ter Haar et al. generated a type of Kapton bellows for use as a heat exchanger by gluing dozens of sheets together [42], but a bellows formed in this manner would be extremely delicate: repeated actuation would degrade the glue joints over time. A seamless Kapton tube would be leak-tight but inextensible, especially at cryogenic temperatures. Crisply folded bellows are commercially available, but these are folded from a flat sheet and bonded along one seam; this joint tends to fail and leak during cryogenic actuation. Furthermore, a hexagonally folded bellows is incapable of smooth flat-faced actuation [40,43], leading to large stress concentrations in the sharp creases which in turn cause the rapid deterioration and mechanical failure seen in bellows with crisply folded edges.

#### 3.2.2. Fabrication Methods

Gaussian curvature, *K*, is an intrinsic property of a surface that is preserved by cutting. This explains why it is impossible to generate an undistorted projection of a world map: the globe has K>0, and a flat sheet of paper has K=0. Surfaces with zero Gaussian curvature everywhere are called “developable surfaces,” and include flat sheets, cylinders, and cones. It is possible to impart a small degree of Gaussian curvature to a Kapton sheet, as demonstrated by the target described in [44], but the curvature is extremely small, and the corresponding functionalized structure would be impossibly large. However, Kapton can be thermoset; i.e., the shape forced by external means can be made permanent by applying a certain temperature. In other words, while Kapton cannot really be stretched, it can be folded and the desired resulting geometry should be developable.

**Figure 6:**
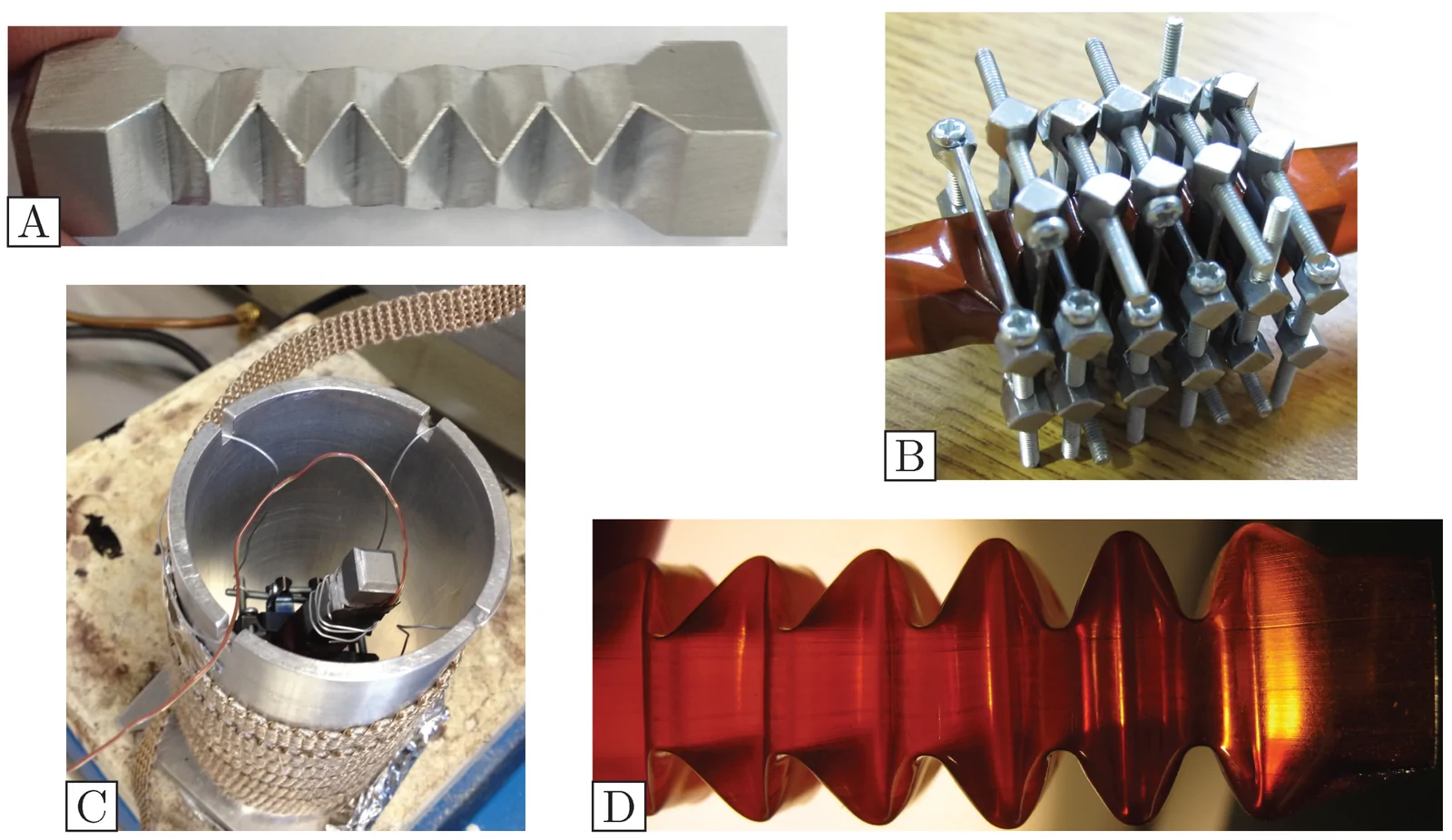
Components used to assemble the bellows. A mandrel that sits inside the tube to guide the folds. Here a 5 cm long mandrel is shown, which was used to fabricate what we called a “short” bellows; later we also used a longer 10 cm version (**A**). The Kapton tube is then clamped onto the mandrel and guided into the correct shape (**B**). Next, the clamped bellows are suspended inside an oven with an internal thermocouple (**C**). After reaching 288 °C, the oven is allowed to cool, and the thermoset bellows can be removed from their mandrel (**D**).

**Figure 7:**
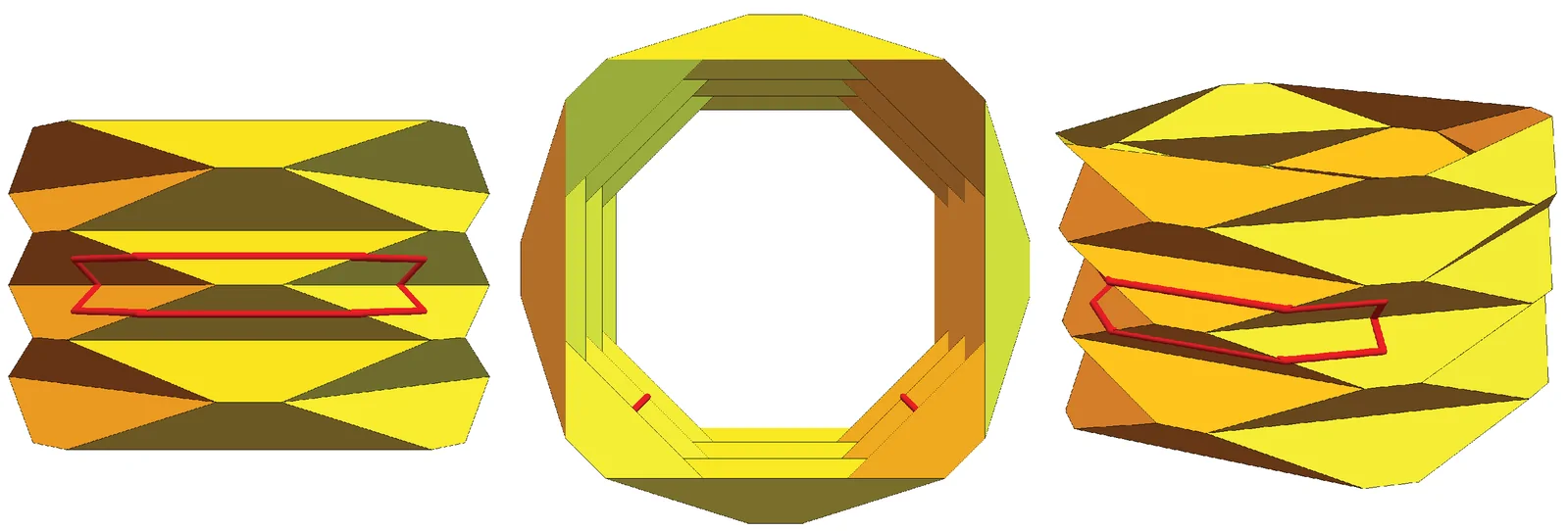
Three views of the hexagonally folded bellow formed by patterning the unit cell outlined in red.

We have developed a set of tools (some are shown in Figure 6) to consistently fold our bellows into the desired hexagonal fold pattern shown in Figure 7. Starting with a machined aluminum mandrel inside the Kapton tube, we applied small clamps so that the tube was pressed into the first groove in the mandrel. For each clamp after the first, while the tube was still able to slide past the mandrel, the extra material was pressed into the crease. This extra material helps smooth out the creases and prevents them from becoming kinked or sharp, as these fold defects generate localized stress concentrations where the film could split when actuated at low temperatures. The clamps were gradually tightened in turn until all were uniformly snug. Next, the clamp and tube assembly were suspended by thin wires in a cool oven, and then gradually heated until the mandrel read 288 °C. Upon reaching the target temperature, we removed the assembly from the heat and allowed it to cool, completing the thermosetting of the Kapton.

After removing the clamps and lubricating the mandrel by pouring a bit of ethanol inside the tube, we pulled the tube from one end (the tube will crinkle like a bendy straw here, which is normal) to unfold and remove it from the mandrel. The mandrel was pressed out of the tube with a smooth rod (we used a chopstick) and the folds in the tube pressed back into place. This process does not introduce leaks into the bellows. Before installing the device, we were careful to inspect all of the folds. Slight variations in fold depth were acceptable, but devices with creases or kinks in the folds themselves will be rigid at low temperatures due to the stress concentration generated at the inadvertently introduced sharp fold.

#### 3.2.3. Scaling Parameters

The bellows that was initially developed and validated used 0.5 in (1.27 cm) diameter Kapton tubing. Extensive cryogenic testing was conducted with this small-diameter bellows. While it proved robust and reliable, its small internal clearance proved unworkable, as the largest rod that the small-OD bellows could accommodate was too weak to reliably actuate. To accommodate a sufficiently strong actuating rod, we scaled the bellows.

**Figure 8:**
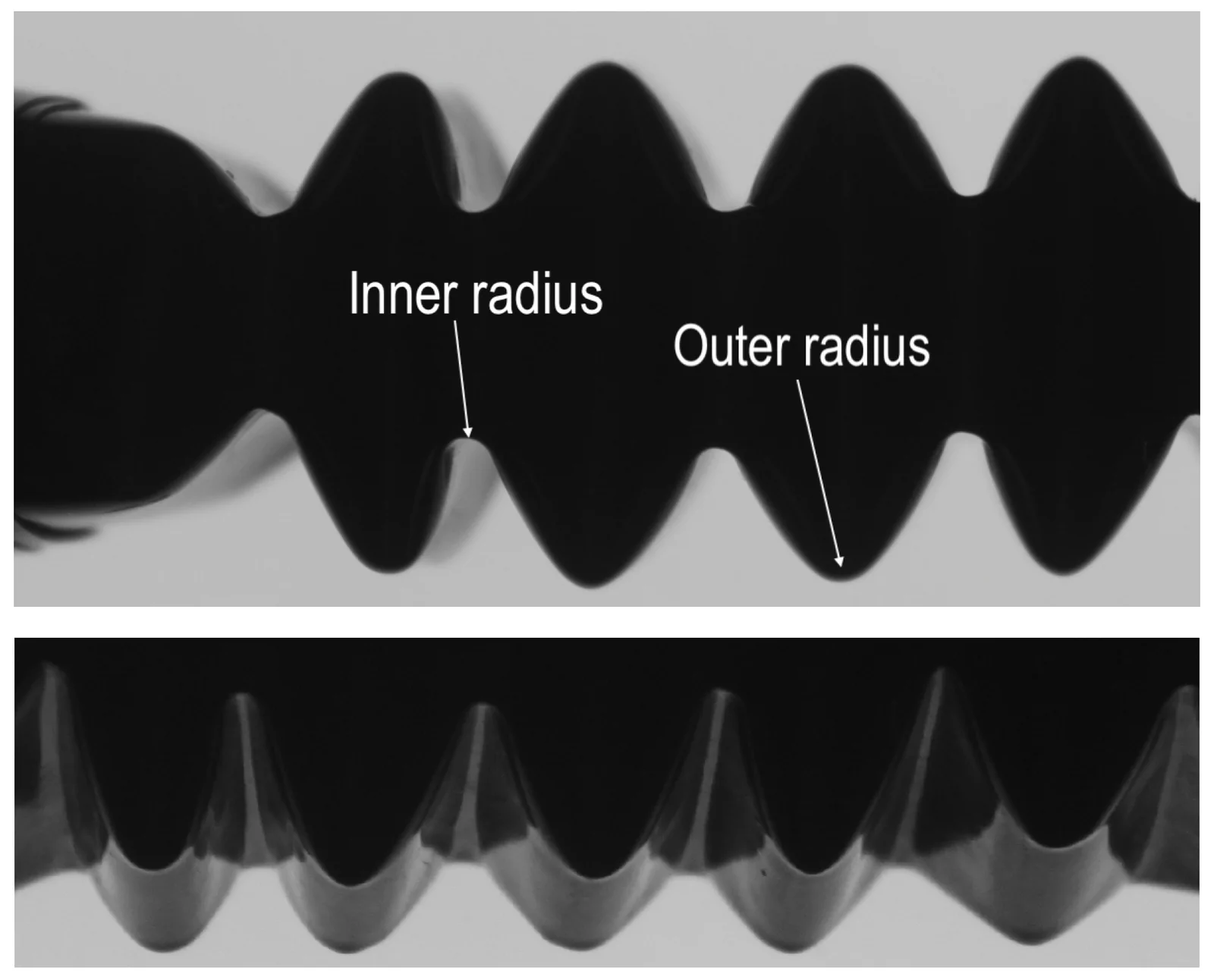
Poorly designed 0.7″ bellows, which proved too brittle. Note that the inner and outer radii of curvature are dramatically different.

Initially, we simply made the bellows 40% larger; this was easily accomplished by directly scaling the engineering model for the mandrel. Scaling the mandrel in all dimensions made the folds both larger and deeper, and the resulting inner creases were far too sharp, as shown in Figure 8.

The irregular folds of Figure 8 clarified which parameters were critical to effectively scale the size of the folded bellows. The method of folding these bellows relies on the substrate’s tendency to bend rather than crease; therefore the local folding/bending interaction should be conserved for a particular wall thickness. The 0.7″ bellows have the same wall thickness as the 0.5″ bellows, so they are successfully folded with the same convolution depth and pitch.

Thinner films are far more resilient to folding, but they are also more permeable to helium at room temperature, complicating leak testing. This bellows’ geometry was empirically determined for a film thickness of around 0.004 inch (0.1 mm). Kapton tubing may be produced in a wide variety of thicknesses, allowing devices with dramatically different wall thickness to be formed with this technique. However, changing the material thickness changes its characteristic bending length [45], so the convolution depth and spacing will need to be adjusted accordingly.

#### 3.2.4. Pressure Resilience and Actuation Durability at Room and Liquid Nitrogen Temperatures

The bellows metamaterial was developed for the actuation of a cryogenic valve. During the warm-up phase of a typical experimental cycle, the bellows needs to be able to withstand elevated internal pressure as the liquid expands and boils. The vacuum system is designed to regularly operate with 1 bar of differential pressure, so the bellows needs to withstand at least 2 bar. The expected maximum number of actuation cycles does not exceed 1000.

We tested the device in stages. First, the pressure resilience and durability of the bellows were tested at room temperature. Next, the bellows was mounted into cryogenic fixtures that allowed actuation in a liquid nitrogen bath and were suitable for superfluid liquid helium leak checking. Both the room-temperature and liquid nitrogen actuator tests were conducted with an Instron 5944, a commercially available system that combines a precise linear actuator, a digital force gauge, and control/logging software.

**Figure 9:**
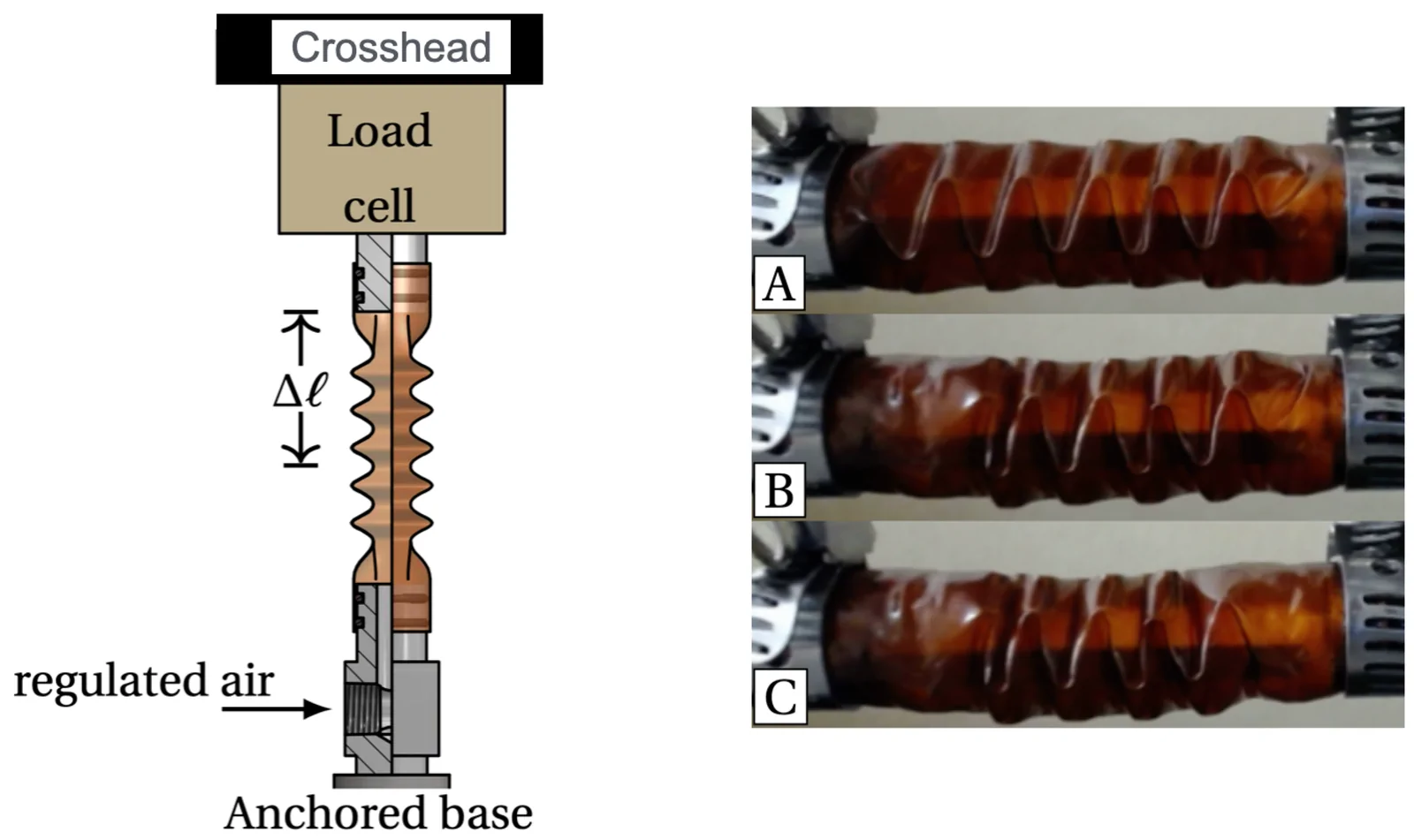
Pressure resilience testing. (**Left**): Room-temperature test setup. The bellows are attached by hose clamps and sealed with double piston seals. The top fixture, which attaches to the Instron’s cross-head, is blank while the lower fixture, which attaches to the Instron’s base, is connected to a pressure regulator. (**Right**): Progressive unfolding of bellows due to internal pressure (differential). (**A**): 11 kPa, (**B**): 258 kPa, (**C**): 305 kPa.

In order to determine the amount of differential pressure a folded bellows can withstand without failure, a short 0.5″ bellows was mounted on the piston-sealed endcaps, drawn in Figure 9, with the test frame locked in place. Because the bellows is sealed on both ends, the internal pressure may be extracted from the load cell attached to one end. By gradually increasing the internal pressure, we were able to observe the primary failure modes of the bellows. Without bracing, a new bellows will resist 125 kPa internal pressure before squirming. Squirm events rapidly fatigue the polymer bellows, and it is far less resistant to repeated tests: failure was observed at 127 kPa, 97 kPa, 75 kPa, and 81 kPa. With an internal support rod, the squirm mode is suppressed and, as illustrated in Figure 9, the bellows accommodates the internal gas pressure by incrementally unfolding from the ends, pressing the remaining folds towards the center. The tubing withstood pressures of 305 kPa without rupturing, but should those pressures be encountered while operating, the bellows will need to be replaced.

During room-temperature compression durability tests, the compressed air pressure was set slightly higher than atmospheric (approximately 1.3 bar absolute) and the bellows was subjected to the periodic linear compression movement by the Instron machine. The pressure is used to detect a rupture, which manifests in a change in the detected load: if the bellows ruptures, the force to move the bellows drops. A 0.5″ diameter bellows was subjected to the room-temperature actuation durability test schedule detailed in Table 3.

At least 1000 cycles of the the 12 mm test were completed, but some data was lost due to computer failure so the total number of cycles is a guess. While testing at 15 mm compression (43% Δℓ/ℓ), failure was observed after 260 cycles. This failure first manifested when the bellows was uncompressed, as compressing the device presses the cracks closed. Subsequent compressions caused these cracks to grow, further decreasing the pressure observed inside the bellows.

Similar tests were conducted on a first-generation 0.7″ bellows similar to that shown in Figure 8. The results are detailed in Table 4. Several bellows split when actuated under pressure, and these failures were attributable to both overbaking the Kapton and less resilience due to the improperly scaled folding pattern. The undamaged bellows could be tested by altering the testing procedure somewhat: instead of actuating the bellows under pressure and waiting for a pressure drop to indicate a failure, the bellows was actuated without a pressure differential, and then pressurized with 5 psi of LN boil-off to check for major leaks.

**Figure 10:**
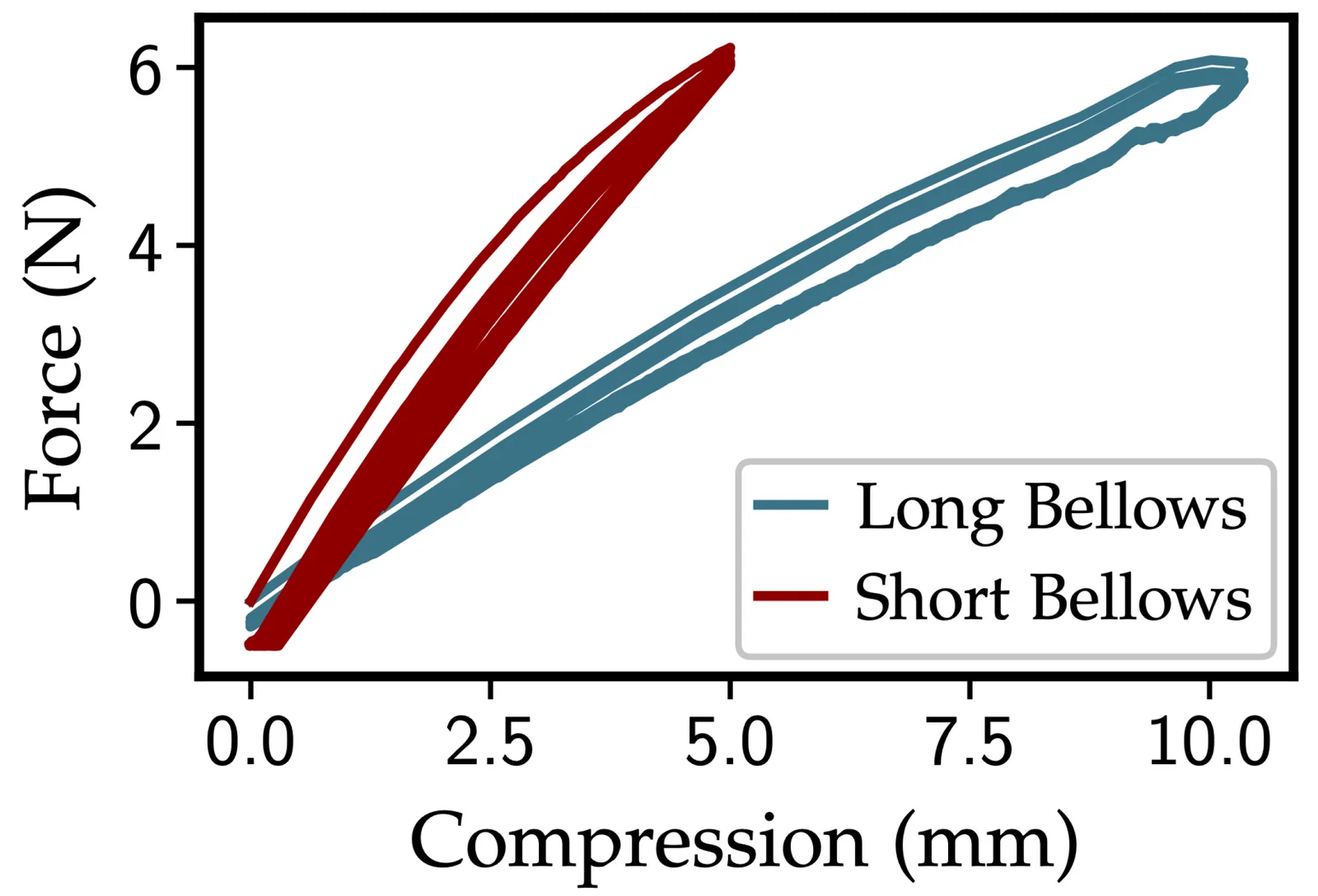
Force–displacement plot of short and long 0.7″ bellows. Note that the long bellows has twice as many convolutions, is twice as long, and has half the spring constant of the short bellows.

Both bellows were tested for Δℓ/ℓ≈13%. The “short” (11 total folds) 0.7″ bellows survived 500 cycles of 5 mm compression at 0.5 mm/s, while the “long” (22 total folds) bellows survived substantially more: 1000 cycles of 0.5 cm compression followed by 1000 cycles of 1 cm compression. As shown in Figure 10, both devices are fairly linear in their spring response, exhibiting a slight softening nonlinearity. The long bellows is twice as long as the short, so it is not surprising that it has half the spring constant.

**Figure 11:**
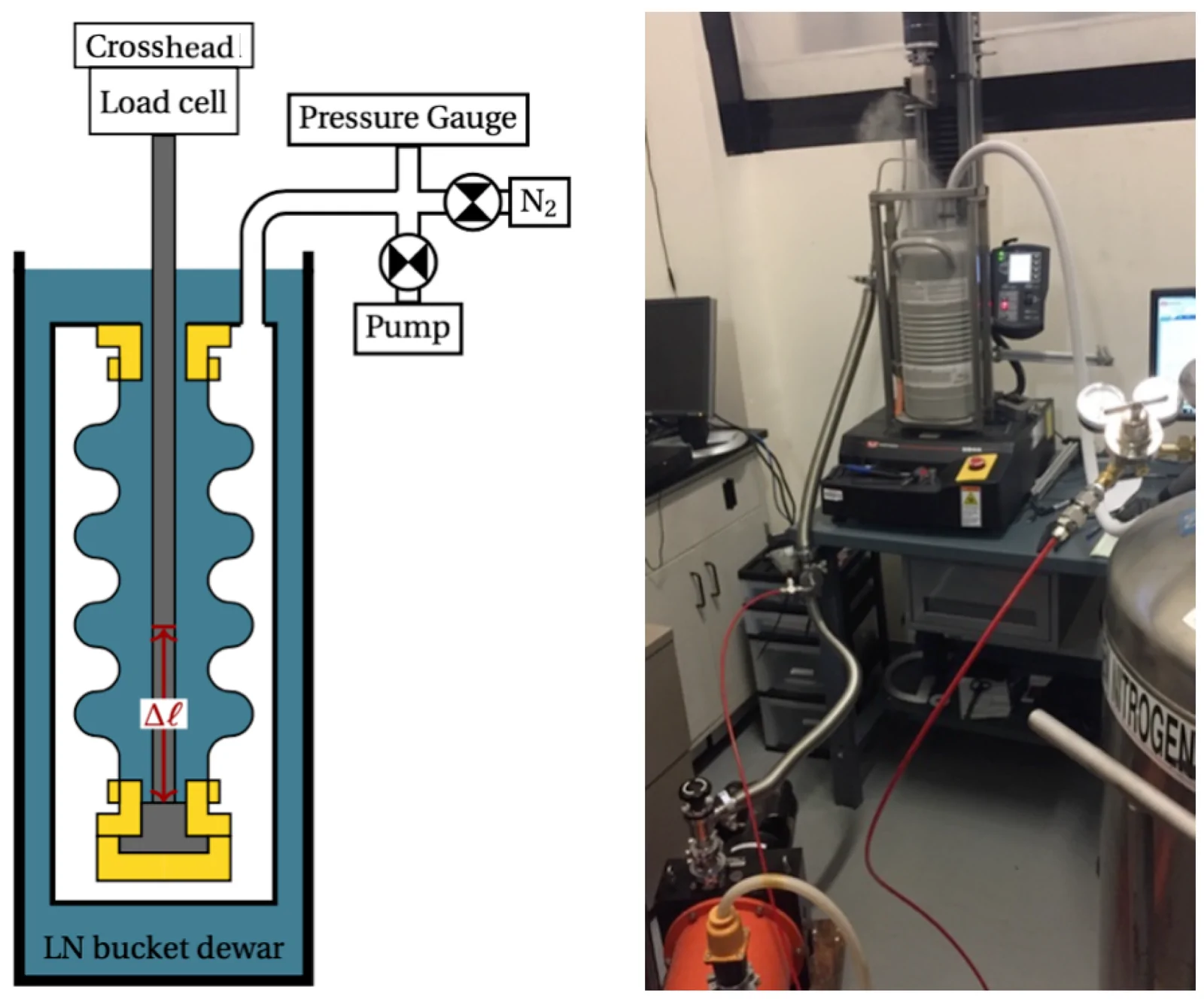
Leak testing during the cryogenic actuation durability test.

Having seen promising room-temperature results, we designed a special setup for cryogenic compression durability testing in liquid nitrogen ($LN_{2}$). As shown in Figure 11, the bellows was mounted on small flanges to be able to make a leak-tight cryogenic assembly. The flanges were made from polyether ether ketone (PEEK) plastic, which is a good non-magnetic mechanical replacement for metal. Stycast 1266 epoxy was used to glue the bellows to the PEEK parts. All gluing surfaces (PEEK and Kapton alike) were scuffed tangentially around their openings with Scotch-Brite before being rinsed, first with alcohol, and then with DI water. The bellows and endcaps were assembled, and Stycast applied to the seam between the two. The bellows assembly was mounted in a small vacuum can. After the epoxy cured, the vacuum can was assembled with indium gaskets and mounted in a 5 L bucket Dewar. This geometry allowed $LN_{2}$ to reach and cool the interior of the bellows, and loaded the bellows with a full atmosphere of internal pressure.

Tests were conducted separately for the 0.5″ and 0.7″ bellows. The 0.5″ device was actuated at 1 bar differential pressure. The Instron machine was again used to actuate the bellows, this time via a rod extending through the inside of the bellows. In this configuration, the can was evacuated to a rough vacuum, and the bellows was cycled 100 times at a slow rate of 0.5 mm s^−1^ and a total displacement of 5 mm (14%Δℓ/ℓ). A pressure gauge indicated the pressure of the vacuum surrounding the bellows. Should the bellows break, the pressure would spike as the nitrogen vented into the vacuum. At the end of this cycling, the can assembly was moved to a liquid helium dewar for subsequent superfluid leak testing. After determining the bellows was superfluid-tight, the assembly was returned to the Instron for more cold cycling. The previous test compressions and rates were used again for 410 cycles more without measurable failure of the bellows.

The 0.7″ bellows testing regimen was modified to more accurately reflect the actual pressure of the bellows in the nEDM test-bed cryostat. Namely, the device needs to withstand static pressurization of 1 bar during normal operation (so 2 bar for safety), but it does not need to support a pressure differential while actuating. This complicated the test setup somewhat: the test can was evacuated to a rough vacuum while the system was cooled by LN, but the can was repressurized with pure nitrogen to prevent oxygen or water from condensing in the can or on the bellows. A more substantial test frame was also designed, allowing substantially more force to be exerted on the bellows without worry of damaging the test can’s vacuum line.

We also tested the 0.7″ bellows at a faster actuation rate. Testing was conducted in 100-cycle increments: a bellows was compressed by 13% (5 mm) at 10 mm s^−1^, held compressed for 2 s, returned to $\ell_{0}$ at −10 mm s^−1^ and held for 2 s to relax. After 100 cycles of this, the vacuum can was evacuated again, checking for major leaks in the bellows. The can was then refilled with 1 bar of nitrogen, and the process was repeated 5 times. A leak from the liquid bath into the vacuum can was observed at this point (implying a 400–500-cycle count lifetime), so testing was terminated.

The measured durability for the 0.7″ bellows and the 0.5″ bellows was substantially different. The 0.5″ bellows withstood nearly 1000 slow compression cycles under vacuum, but the 0.7″ bellows only withstood 400 fast compressions without a pressure differential. It is not clear whether the 0.7″ bellows is globally less resilient than the 0.5″ design, or if rapid actuation (10 mm/s vs. 0.5 mm/s) is more fatiguing, but it is most likely a combination of the two effects. These tests were conducted with compression and extension rates of 10 mm/s, with only two seconds of dwell time between actuations. These are far more challenging conditions than the proposed operating cycles of the nEDM test-bed experiment. Subsequent tests should be conducted with longer pauses and a slower relaxation rate, which should substantially improve the lifespan of the bellows. Nevertheless, the Kapton bellows showed substantial promise as a non-magnetic and RF-transparent metamaterial that can be used for valve actuation in the nEDM test bed.

### 3.3. Superconducting Joint

Setting a limit on the nEDM requires searching for extremely small frequency shifts in the Larmor precession of the observed neutrons [8,12]. This means that fluctuations or drifts in the $B_{0}$ magnetic field—which would change the precession frequency—must be minimized accordingly. For the stated aim of measuring $d_{n}$ ≤ 3 × 10^−28^ e·cm [10], we need to ensure that the magnetic field has a temporal drift of less than 1 part per 10 billion per second, a level of stability that cannot be achieved by any power supply.

In a cryogenic experiment, we can meet this requirement by running our superconducting (SC) $B_{0}$ magnet coil in persistent current mode. The idea is quite simple: a circuit that is completely SC will have zero resistance, and therefore any current that is induced in it will flow “forever”; in practice, one simply ensures a maximum circuit resistance that will ensure that the desired stability is met (this is $R<10^{-13}\;\Omega$ for nEDM@SNS [46]). In order to induce (via an external magnetic field or power supply) and then hold the desired current, the circuit can be “opened” and “closed” by bringing a portion of the wire above and below the critical temperature. This is done by inserting a heat switch into a portion of the circuit. Finally, to complete the circuit, the magnet coil and the heat switch must be connected by superconducting joints. These joints are the most likely cause of resistance in the final circuit and can be tricky to make, especially in experiments like cryogenic nEDM that have very specific magnetic, cryogenic, and material constraints. For this reason, we developed a novel technique for creating SC joints that we believe will allow us to consistently transfer our heat switch across our different experimental apparatuses.

#### 3.3.1. Design Requirements

A fully realized superconducting loop will be made of SC wire connected in at least one location by an SC joint. In experiments like ours that induce the field via an external power supply, two joints are needed. SC wire is commercially available and well characterized [47,48,49]. SC joints, on the other hand, must be made after the coil is wound.

For a highly specialized application such as our nEDM test bed, we needed to create joints that met our magnetic field and current requirements, could be reliably made in small, open-air environments, and were transferable across several coils in the testing regime for the nEDM@SNS experiment. In order to meet the minimum stability requirements of our $B_{0}$ coil, the SC joints must contribute a resistance of less than $10^{-13}\;\Omega$ to the final circuit while maintaining a current of up to 500 mA. Additionally, all of the materials used in the construction of the joints must be non-magnetic ($\left|\chi_{\nu }\right|<10^{-4}$ [50,51,52]). The existing design of the nEDM test-bed apparatus required us to keep the footprint of the final heat switch and joints small, around 25 cm^2^ or less. At the same time, we hoped to make a single SC switch that could be transferred from a test apparatus to the nEDM test bed to nEDM@SNS with a joint procedure that could be easily and safely replicated in the open-air environment present during commissioning.

Several methods for creating SC joints have been developed over the years (see [11] for a comprehensive overview) for now-common applications like high-current SC magnets used in magnetic resonance imaging [53,54] (which usually operate in the 10–100 A range) and single-filament low-current SC joints in SQUIDs [55] (nAs to hundreds of μA). Stringent requirements must be met when a coil is to be operated in persistent mode. Of primary concern is the tendency for niobium (Nb)—the most common superconductor used in these applications—to oxidize when exposed to air [56], thereby leaving some resistive oxide on the wire surface and interrupting the superconductivity of the loop. Each method addresses this problem slightly differently. Diffusion bonding [57] and spot welding [58] were not seriously considered for our application due to their difficult manufacturing procedure and inconsistent joint performance. We attempted to create several joints using the cold press method [59,60], in which the Cu wire matrix is stripped with nitric oxide (HNO_3_) and the exposed Nb filaments are then rinsed with hydrofluoric acid (HF) before bring crushed together using a press. We also built several using the Thornton matrix replacement method [61,62], where the Cu matrix is removed via a bath of molten Sn and replaced with molten SC solder (of which several are commercially available) before the wire ends are twisted together as the solder cools. Both of these techniques showed promise for our experiment, as several test joints were created and proven to be superconducting.

However, we struggled to consistently produce successful joints and were hesitant to use a highly corrosive chemical (HF in the cold press method) or high-temperature molten metals (Sn and solder baths in the matrix replacement method) as we attempted to build our final joint in situ with our wound $B_{0}$ coil out of concern for damage to the existing experiment or researcher. Unlike higher-current applications, we do not need to maximize the critical current of the joints. Therefore, we aimed to create a procedure that was simpler, safer, and more consistent to implement.

#### 3.3.2. Joint Method

Ultimately, we decided to combine the experience from the procedures described above with a simple press method used to make low-current SC circuits in SQUIDs between the external SC pickup loop and the SQUID input coil [63]. To close the SQUID circuit, oxides are first removed from the single-filament NbTi wire via a razor blade and from the small Nb surface via a fiberglass brush, and the two are then pressed together using only a brass screw. Because the SQUID pickup loop operates in the nano- or micro-amp range [64] and is only a single filament wire, however, it remained an open question if such a simple connection would suffice for our multi-filament and higher-current (500 mA) application.

Unlike traditional SC joining methods, we chose to connect the filaments of the separate wires to a common piece of Nb (we therefore refer to our procedure as “the Nb pad method”). While this doubles the number of SC connections that could possibly add resistance (two for the switch–Nb connection and two for the coil–Nb), it also allows us to independently join the switch and the coil, allowing us to more easily transfer the switch between different magnets.

**Figure 12:**
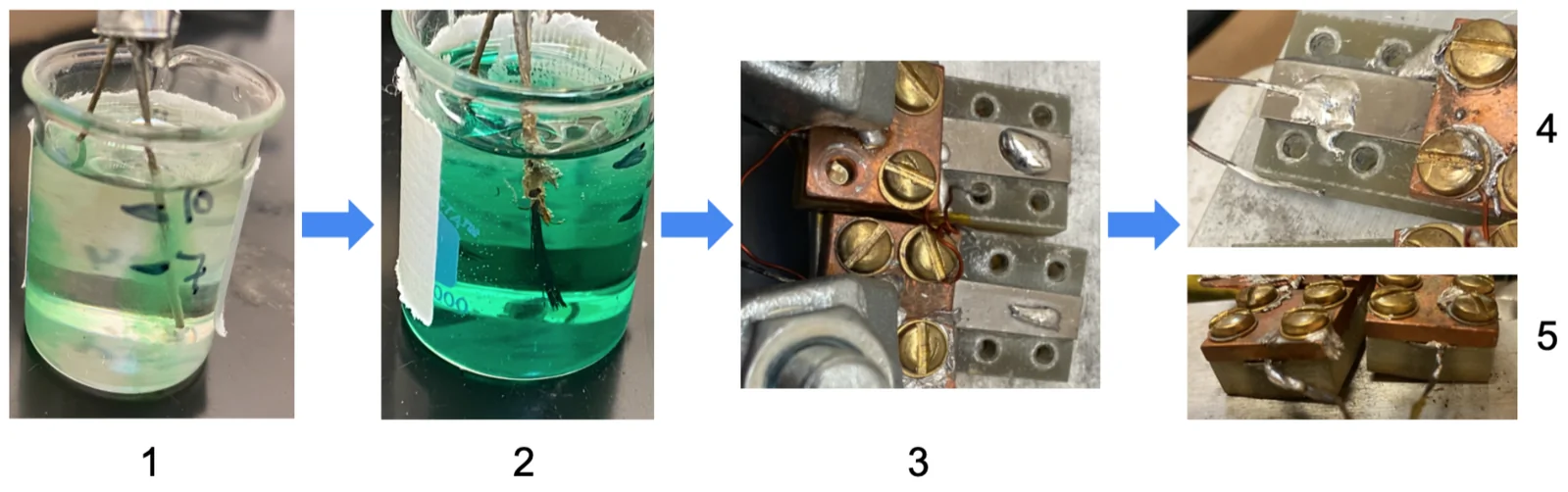
A new SC joint method that combines elements from previous press and solder joining methods. After stripping the SC wire of insulation, the Cu-matrix is etched away with HNO_3_ for two hours (**1**). When the NbTi filaments are exposed (**2**), they are removed from the solution, rinsed with deionized water, placed (**4**) onto a warmed (∼110 °C) and deoxidized (fiberglass brush) Nb surface with a small amount of molten Ostalloy203 (**3**), and then pressed with a Cu washer (**5**). For dimensions of the joints, see the text.

Figure 12 shows the implementation of the Nb pad method for multi-filament wires (the same type of wire—54S43 NbTi from Supercon Inc. (Shrewsbury, MA, USA)—will be used for both our heat switch and our $B_{0}$ coil) in an open-air environment. First, the NbTi filaments of the SC coil wires were exposed by dissolving the stabilizing Cu matrix in 50% nitric acid (HNO_3_) for two hours. As the two-hour mark approached, the 0.7 × 3.8 cm Nb strips—epoxied with Epo-Tek T7109-19 onto an electrically insulating G-10 base (2.1 × 3.8 cm)—were heated to ∼110 °C on a hot plate. Then, just before the now-exposed filaments were removed from the acid bath, we used a fiberglass brush to scrape the oxides off the surface of the elemental Nb and added a drop of molten SC solder (15.5Sn–32Pb–52.5Bi, known as Ostalloy203) to the Nb strip to further improve the SC connection between the filaments and the Nb pad. Next, the visible filaments were removed from the nitric acid solution, rinsed with deionized (DI) water, and quickly placed (≤5 min) onto the Ostalloy droplet on the Nb pad. Finally, the connection was secured to tapped holes in the G-10 base with an oxygen-free (OFHC) Cu washer and brass screws. Overall, this procedure takes less than 150 min of total time (and less than 20 min of working time), temperatures no higher than 110 °C, no dangerous HF, and tools as simple as a couple of clamps, a hot plate, and a screwdriver, making it appealing for implementation in our experiment.

#### 3.3.3. Superconducting Tests

After the joints were constructed, we needed to confirm that they were actually superconducting. A common way to do this is to measure the resistance of the circuit via inductive resistance testing (IRT), also known as the current (or field) decay method. First introduced by Leupold and Iwasa [65], IRT utilizes the simplicity of a superconducting loop by modeling it as an LR circuit with an extremely long time constant (a valid approximation for passable SC joints). The resistance can then be extracted from a measurement of the current—or magnetic field—decay. The slope of the decay is related to the resistance of the SC circuit as

(5) $$R=\left|\frac{\delta B}{B}\right|\;\frac{L}{\Delta t},$$

where *t* is the measurement time, *L* is the coil inductance, and $\left|\delta B/B\right|$ is the ratio of the decay to the magnitude of the field.

**Figure 13:**
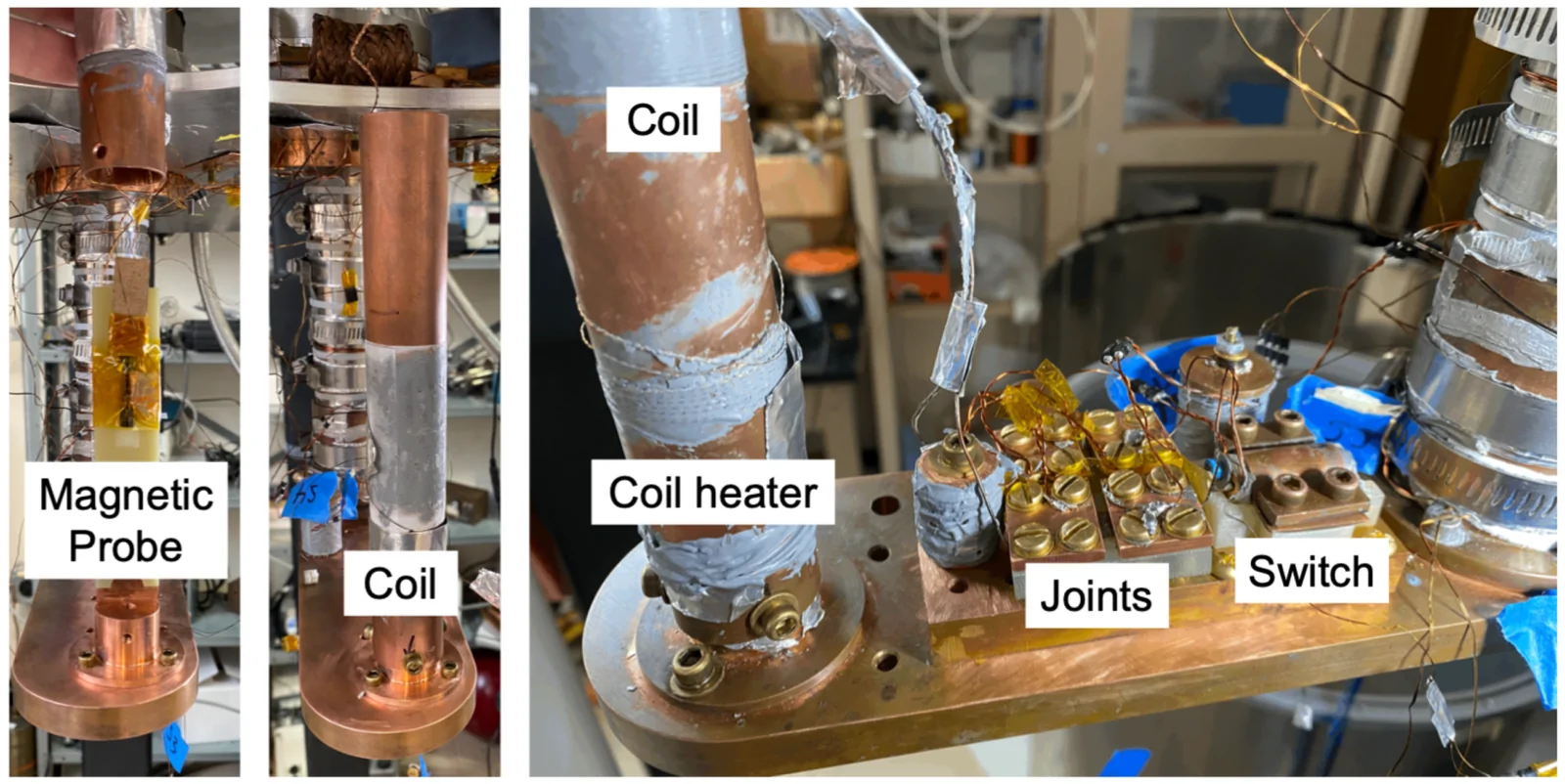
The cryostat setup for a integrated superconducting joint and switch test. A fluxgate magnetometer (**first panel**) measures the axial field at the center of our test SC coil (**second panel**). The coil is connected to the heat switch via the Nb pad superconducting joints, onto which the current leads are soldered (**third panel**).

Our joints, test SC coil (30 turns, 2.9 cm diameter, and 1.8 cm long with a measured inductance, *L*, of (5.6±0.4) μH), and (when applicable) heat switch were assembled on the second stage of the RDK-15 cryocooler (the same as in Section 2.2.2). The assembly is shown in Figure 13. A Bartington fluxgate magnetometer (Mag-01H with an axial Mag F probe) was placed at the center of the SC coil to measure the current/field of the circuit. For single-joint tests—in which the two ends of the coil are closed via one joint and not attached to the heat switch or external supply—an external “inducing coil” was placed outside the cryostat and coaxial to the SC coil to change the magnetic field passing through the circuit. For finer current control during complete circuit tests with the heat switch attached, Cu wires were soldered to the Cu washers to connect the circuit to a custom external power supply [66]. Heater wire (36-AWG Phosphor Bronze) was used to independently control the temperature of the SC coil and the heat switch. Temperature sensors (DT-670 diodes from Lakeshore Cryotronics) were attached to the coil, joints, switch, and various points along the cryostat to monitor each of the components, particularly as the superconductors passed their critical temperatures ($T_{c}$).

The very first time we tried the Nb pad method—with the common strip of Nb and filaments secured via the Cu washers—we exposed the NbTi filaments with Sn and solder baths at ∼320 °C (for 150 and 60 min respectively), following the initial steps of the matrix replacement method. When the filaments of both ends of the coil were ready, they were secured to either end of the Nb pad with the Ostalloy droplet and Cu washers and screws. The coil and joint were then placed in the cryostat.

As the circuit was cooled, we observed a superconducting transition—as evidenced by a minor field change followed by a “trapping” of magnetic flux—at 8.7 K, which is the $T_{c}$ of the Ostalloy203 solder [11]. This was an early indication that the SC current is not passing directly from the NbTi filaments to the Nb strip (both have a $T_{c}$ of 9.2 K), but through the solder. It was beyond the scope of our work to confirm this assumption by taking detailed microscopic images, but this would align with previous observations of SC solder/filament connections [67]. The supercurrent held by our coil and initial Nb pad joint was calculated to be 1.2 ± 0.1 mA and shown to be stable for hundreds of seconds while the circuit was superconducting. The final $B_{0}$ coil will operate at hundreds of mAs for thousands of seconds, necessitating a second iteration of joint production and testing, but these initial results proved that we could incorporate methods from other SC joint procedures to create a circuit that met all the production and operational requirements of our experiment.

Having demonstrated the general concept of our custom joint method, we created a second set of joints following the full Nb pad procedure, this time exposing the NbTi filaments with the nitric acid solution. The two ends of the coil were connected to separate joints, with the other end of each Nb pad securing the NbTi filaments from the heat switch (and current leads soldered to the washers). Once the circuit was cooled, a current of 450 mA (measured as a (303.8±0.5) μT magnitude field on the magnetometer) was generated in the circuit via our external power supplies and the heat switch was closed, trapping the supercurrent. This current was maintained for $9\times 10^{4}$ s with extremely little decay; linear regression was used to determine the slope of the decay (δB/Δt) to be (2.937±0.002) × 10^−7^ μTs^−1^. Using Equation (5), we determined that the resistance of our circuit was $R=\left(5.6\pm 0.4\right)\times 10^{-15}\;\Omega$, corresponding to a 95% confidence interval of $R\leq 6.2\times 10^{-15}\;\Omega$ for the entire circuit (and therefore $R\leq 3.1\times 10^{-15}\;\Omega$ per joint). This is more than sufficient to achieve the desired temporal stability for nEDM@SNS, and proves that the Nb pad method is a viable one for producing joints that operate up to 500 mA. Because this is a novel joining technique, future performance will be monitored closely to determine the long-term stability of the joints, particularly if new ones are made during potential coil changes. A full discussion of the performance of the heat switch is beyond the scope of this paper, but more details can be found in [46] and an upcoming paper on this component.

## 4. Conclusions

We have presented results of four unique material developments motivated by fundamental physics research with neutrons: two cryogenic metamaterials (one thermal and one mechanical) and two combinations of materials never used in cryogenics before. We described their design, fabrication methods, and results of thermal and mechanical testing under cryogenic conditions.

First, we demonstrated the design and experimental verification for the first-of-its-kind thermal metamaterial fabricated by additive manufacturing from Ti6Al4V alloy powder using Arcam AB’s EBM S12 system. The material was designed to have a specific thermal conductivity geometric factor, *g*-factor, and be slightly flexible. We also introduced an easy technique to measure experimental *g*-factors; incrementally increasing the heater power allowed us to estimate the experimental *g*-factor to within about 10%, with much of that value coming from inconsistencies in the literature thermal conductivity data. Experimental *g*-factor values show fair agreement with the simulated ones. For practical cryogenic design, we recommend using a safety factor ≃ 2 or above. Note that our samples were built with an old EBM system. With modern EBM machines, better electron-beam parameter control might be possible, resulting in better quality of nominal 0.7 mm strut fabrication for smaller *g*-factors.

We conclude that EBM-built Ti6Al4V lattice metamaterial can be successfully used as a thermal spacer and mechanical support in cryogenic designs with limited distance between warm and cold surfaces. These supports performed well with no signs of degradation over many thermal cycles during our solid deuterium and methane studies.

Another innovative cryogenic material is the Zircaloy-4 alloy explosively bonded to an Al6061 plate. Zircaloy-4 is a low-thermal-conductivity material attractive for its high radiation resistance and low neutron absorption cross-section. Al6061 alloy is a high-thermal-conductivity alloy also widely used in neutron-related applications. While Al6061 is a common choice for cryogenic parts in nuclear reactors, Zircaloy-4 is typically used and tested at high temperatures. We report the first successful cryogenic use of Zircaloy-4 explosively bonded to Al6061 as a temperature-gradient bridge between two Al6061 parts of the cryostat. Such a design can be very useful for future cryogenic neutron sources.

For low- and extremely sensitive magnetic field environments, we report the use of a first-of-a-kind origami plastic metamaterial for actuating a valve immersed in superfluid LHe. We have developed a method of folding a seamless Kapton tube into a flexible bellows. At room temperature the bellows withstood more than 2000 actuations. We also report details of making superfluid tight joints to PEEK flanges so that the bellows assembly is capable of superfluid leak-tight cryogenic actuation. A sample bellows withstood 500 cycles of fast compression by 5 mm (14% Δℓ/ℓ) in liquid nitrogen. We believe that with slower actuation the result can be improved. While it is not as resilient as a conventional beryllium copper (BeCu) device, this folded bellows is both RF-transparent and non-magnetic.

In order to operate the superconducting coil in persistent current mode, we needed to create superconducting joints to connect the coil to the power supply and a superconducting heat switch. Previous superconducting joint applications include high-current (>10 A) superconducting magnets and low-current (<100 μA) single-filament wires. We developed a new method for creating multi-filament superconducting joints that operate in a moderate current range: up to 500 mA. This new process is both easier to implement than older methods for higher-current applications and allows joints for individual coils to be exchanged without impacting the connection to the heat switch or power supply. This is useful for the development and testing of different coils during cryogenic commissioning. We demonstrated that the resistance of these joints does not exceed the $10^{-15}$ Ω range, meeting the temporal stability required for our cryogenic nEDM experiment.

## Figures and Tables

**Table 1:** Combined experimental data for Samples 1–4 containing the sample number, top heater power, bottom and top temperatures, and $g_{\exp}$-factor.

Sample	$P_{\mathrm{top}}$ (mW)	$T_{\mathrm{bottom}}$ (K)	$T_{\mathrm{top}}$ (K)	$g_{\exp}$ (mm)
**Sample 1: 4 × 6 × 4 cells, 1.0 mm**				
1	0	36.6	58.8	N/A
1	1.78	36.0	60.2	0.49
1	3.50	36.2	61.6	0.55
1	5.77	36.8	63.3	0.52
**Sample 2: 4 × 6 × 4 cells, 0.7 mm**				
2	0	34.8	76.5	N/A
2	1.6	35.1	78.5	0.30
2	3.1	35.5	80.1	0.30
2	5.1	35.9	82.2	0.30
**Sample 3: 4 × 4 × 4 cells, 1.0 mm**				
3	0	29.3	52.4	N/A
3	1.81	29.7	54.3	0.39
3	3.57	30.2	56.3	0.36
3	4.89	30.6	57.9	0.37
**Sample 4: 4 × 4 × 4 cells, 0.7 mm**				
4	0	30.2	69.1	N/A
4	1.85	32.3	73.7	0.20
4	3.67	32.8	76.6	0.21
4	6.02	33.3	80.1	0.21

**Table 2:** Lattice dimensions, measured and simulated *g*-factors, and the ratio $g_{\exp}/g_{\mathrm{sim}}$

Sample	L × W × H Cells	Strut (mm)	$g_{\exp}$ (mm)	$g_{\mathrm{sim}}$ (mm)	Ratio
1	4 × 6 × 4	1.0	0.52	0.40	1.30
2	4 × 6 × 4	0.7	0.30	0.19	1.56
3	4 × 4 × 4	1.0	0.37	0.27	1.37
4	4 × 4 × 4	0.7	0.21	0.13	1.61

**Table 3:** Room-temperature compression durability test schedule for 0.5″ diameter short (5 cm) bellows.

Δl (mm)	Δl/l (%)	Cycles
3	8.6	278
6	17.1	300
9	25.7	300
12	34.2	1000–2000
15	43.0	260

**Table 4:** Room-temperature fatigue tests for short (5 cm) and long (10 cm) 0.7″ diameter bellows. Bellows integrity was tested after every 100 test cycles. The same long bellows was subjected to all the tests reported here.

*l* (mm)	Δl (mm)	Δl/t (mm/s)	Δℓ/ℓ (%)	Cycles
38.1	5	0.5	13.1	1700
76.2	5	0.5	6.6	1000
	10	0.5	13.1	500
	10	10	13.1	500

## Data Availability

The original contributions presented in this study are included in the article. Further inquiries can be directed to the corresponding author.

## Abbreviations

The following abbreviations are used in this manuscript:

UCNs	Ultra-cold neutrons
nEDM	Neutron electric dipole moment
SNS	Spallation Neutron Source
SC	Superconducting
SQUID	Superconducting quantum interference device
Zry-4	Zircaloy-4 alloy
EB	Electron beam
EBM	Electron Beam Manufacturing

## References

[1] Michaudon A. (1997). Use of Ultracold Neutrons for Condensed-Matter Studies.

[2] Golub R. (1996). Ultracold neutrons: Their role in studies of condensed matter. Rev. Mod. Phys..

[3] Golub R., Pendlebury J. (1975). Super-thermal sources of ultra-cold neutrons. Phys. Lett. A.

[4] Anghel A., Atchison F., Blau B., Van den Brandt B., Daum M., Doelling R., Dubs M., Duperrex P.A., Fuchs A., George D. (2009). The PSI ultra-cold neutron source. Nucl. Instrum. Methods Phys. Res. Sect. A Accel. Spectrometers Detect. Assoc. Equip..

[5] Ito T., Adamek E., Callahan N., Choi J., Clayton S., Cude-Woods C., Currie S., Ding X., Fellers D., Geltenbort P. (2018). Performance of the upgraded ultracold neutron source at Los Alamos National Laboratory and its implication for a possible neutron electric dipole moment experiment. Phys. Rev. C.

[6] Martin J., Franke B., Hatanaka K., Kawasaki S., Picker R. (2021). The TRIUMF UltraCold advanced neutron source. Nucl. Phys. News.

[7] Chanel E., Baudoin S., Baurand M.H., Belkhier N., Bourgeat-Lami E., Degenkolb S., van der Grinten M., Jentschel M., Joyet V., Kreuz M. (2022). Concept and strategy of SuperSUN: A new ultracold neutron converter. J. Neutron Res..

[8] Golub R., Lamoreaux S.K. (1994). Neutron electric-dipole moment, ultracold neutrons and polarized 3He. Phys. Rep..

[9] Abel C., Afach S., Ayres N.J., Baker C.A., Ban G., Bison G., Bodek K., Bondar V., Burghoff M., Chanel E. (2020). Measurement of the permanent electric dipole moment of the neutron. Phys. Rev. Lett..

[10] Ahmed M., Alarcon R., Aleksandrova A., Baeßler S., Barron-Palos L., Bartoszek L., Beck D., Behzadipour M., Berkutov I., Bessuille J. (2019). A new cryogenic apparatus to search for the neutron electric dipole moment. J. Instrum..

[11] Brittles G.D., Mousavi T., Grovenor C.R.M., Aksoy C., Speller S.C. (2015). Persistent current joints between technological superconductors. Supercond. Sci. Technol..

[12] Cianciolo V., Golub R., Filippone B., Huffman P., Leung K., Korobkina E., Swank C. (2024). The Systematics and Operational Studies (SOS) Apparatus as a testbed for nEDM@ SNS experiment. arXiv.

[13] Korobkina E., Medlin G., Wehring B., Hawari A., Huffman P., Young A., Beaumont B., Palmquist G. (2014). Ultracold neutron source at the PULSTAR reactor: Engineering design and cryogenic testing. Nucl. Instrum. Methods Phys. Res. Sect. A Accel. Spectrometers Detect. Assoc. Equip..

[14] Medlin G., Korobkina E., Teander C., Wehring B., Sharapov E., Hawari A.I., Huffman P., Young A.R., Palmquist G., Morano M. (2024). External moderation of reactor core neutrons for optimized production of Ultra-Cold neutrons. J. Nucl. Eng..

[15] Golub R., Pendlebury J. (1977). The Interaction of Ultra-Cold Neutrons (UCN) with Liquid Helium and a Superthermal UCN Source. Phys. Lett..

[16] Ito T.M., Beck D.H., Golub R., Huffman P.R. (2007). Plans for a Neutron EDM Experiment at SNS. J. Phys. Conf. Ser..

[17] Serebrov A., Mityukhlyaev V., Zakharov A., Kharitonov A., Shustov V., Kuz’minov V., Lasakov M., Tal’daev R., Aldushchenkov A., Varlamov V. (2000). Studies of a solid-deuterium source for ultra-cold neutrons. Nucl. Instrum. Methods Phys. Res. Sect. A Accel. Spectrometers Detect. Assoc. Equip..

[18] Kahlenberg J., Ries D., Ross K.U., Siemensen C., Beck M., Geppert C., Heil W., Hild N., Karch J., Karpuk S. (2017). Upgrade of the ultracold neutron source at the pulsed reactor TRIGA Mainz. Eur. Phys. J. A.

[19] Frei A. (2022). The source for ultra-cold neutrons at the FRM II. J. Neutron Res..

[20] Gu J., Zhao W., Zeng C., Liu L., Leng J., Liu Y. (2025). Construction of mechanical metamaterials and their extraordinary functions. Compos. Struct..

[21] Risegari L., Barucci M., Lolli L., Ventura G. (2008). Low temperature thermal conductivity of Ti6Al4V alloy. J. Low Temp. Phys..

[22] (2026). Center, National Nuclear Data Center NuDat 3.0. https://www.nndc.bnl.gov/nudat3.

[23] Younger C.L., Haley F.A. (1969). Effect of Nuclear Radiation at Cryogenic Temperatures on the Tensile Properties of Titanium and Titanium-Base Alloys.

[24] Marmy P., Leguey T. (2001). Impact of irradiation on the tensile and fatigue properties of two titanium alloys. J. Nucl. Mater..

[25] NIST (2026). Material Properties: Ti 6Al 4V (UNS R56400). https://trc.nist.gov/cryogenics/materials/Ti6Al4V/Ti6Al4V_rev.htm.

[26] Eldesouky I., Harrysson O., West H., Elhofy H. (2017). Electron beam melted scaffolds for orthopedic applications. Addit. Manuf..

[27] Cansizoglu O., Harrysson O., Cormier D., West H., Mahale T. (2008). Properties of Ti–6Al–4V non-stochastic lattice structures fabricated via electron beam melting. Mater. Sci. Eng. A.

[28] Ziegler W., Mullins J., Hwa S. (1963). Specific heat and thermal conductivity of four commercial titanium alloys from 20 to 300 K. Advances in Cryogenic Engineering: Proceedings of the 1962 Cryogenic Engineering Conference University of California Los Angeles, Los Angeles, CA, USA, 14–16 August 1962.

[29] Umezawa O., Ishikawa K. (1992). Electrical and thermal conductivities and magnetization of some austenitic steels, titanium and titanium alloys at cryogenic temperatures. Cryogenics.

[30] Kalienko M., Zhelnina A., Volkov A., Leder M., Tolstykh N., Bocharov A. (2020). A New approach to estimation of the thermal conductivity of titanium alloys. Crystallogr. Rep..

[31] Krishnan R., Asundi M. (1981). Zirconium alloys in nuclear technology. Proc. Indian Acad. Sci. Sect. C Eng. Sci..

[32] Nanstad R.K., Server W.L., Kombaiah B., Geringer J.W. Material properties of non-irradiated zircaloy 4 in support of ASME code acceptance for pressure vessel design. Proceedings of the Pressure Vessels and Piping Conference, San Antonio, TX, USA, 14–19 July 2019.

[33] Popov F., Kawalek A., Ozhmegov K., Lutchenko N., Panin E., Lezhnev S., Arbuz A. (2026). Study of the Influence of Thermomechanical Treatment on the Structure and Properties of Zircalloy-4 Alloy. Materials.

[34] Sarkar A., Murty K.L. (2015). Microstructure–mechanical property correlation of cryo rolled Zircaloy-4. J. Nucl. Mater..

[35] Gutsmiedl E., Scheuer A. (2002). Use of Zircaloy 4 material for the pressure vessels of hot and cold neutron sources and beam tubes for research reactors. Phys. B Condens. Matter.

[36] AZOmaterials (2026). Zircaloy-4(Alloy Zr4) (UNS R60804). https://www.azom.com/article.aspx?ArticleID=7644.

[37] Dwivedi D.K. (2023). Dissimilar Metal Joining.

[38] Korobkina E., Berkutov I., Golub R., Huffman P., Hickman C., Leung K., Medlin G., Morano M.J., Rao T., Teander C. (2022). Growing solid deuterium for UCN production. J. Neutron Res..

[39] Wilson M.N. (1983). Superconducting Magnets.

[40] Reid A., Lechenault F., Rica S., Adda-Bedia M. (2017). Geometry and design of origami bellows with tunable response. Phys. Rev. E.

[41] Davidson M., Markley F., Bastian S. (1992). Measurement of the elastic modulus of Kapton perpendicular to the plane of the film at room and cryogenic temperatures. Supercollider 4.

[42] Ter Haar E., Wagner R., van Woerkens C.M., Steel S.C., Frossati G., Skrbek L., Meisel M.W., Bindilatti V., Rodrigues A.R., Martin R.V. (1995). Plastic dilution refrigerators. J. Low Temp. Phys..

[43] Connelly R., Sabitov I., Walz A. (1997). The bellows conjecture. Beitr. Algebra Geom..

[44] Kendellen D., Ahmed M., Baird E., Feldman G., Perreau N., Wallace P., Weller H. (2016). A cryogenic target for Compton scattering experiments at HI*γ*S. Nucl. Instrum. Methods Phys. Res. Sect. A Accel. Spectrometers Detect. Assoc. Equip..

[45] Lechenault F., Thiria B., Adda-Bedia M. (2014). Mechanical response of a creased sheet. Phys. Rev. Lett..

[46] Hickman C. (2025). Development of Magnetic and Neutron Systems Contributing to the Search for the Electric Dipole Moment of the Neutron. Ph.D. Thesis.

[47] Gregory E. (1989). Conventional wire and cable technology. Proc. IEEE.

[48] Wilson M.N. (2008). NbTi superconductors with low ac loss: A review. Cryogenics.

[49] Lyly M., Holm M., Stenvall A., Mikkonen R. (2013). Design Process for a NbTi Wire With New Specification Objectives: Technical Design Constraints and Optimization of a Wire Layout Considering Critical Current and AC Losses. IEEE Trans. Appl. Supercond..

[50] Ekin J.W. (2006). Experimental Techniques for Low-Temperature Measurements: Cryostat Design, Material Properties, and Superconductor Critical-Current Testing.

[51] Collings E.W., Smith R.D. (1972). The magnetic susceptibility of niobium. J. Less Common Met..

[52] Abrecht M., Adare A., Ekin J.W. (2007). Magnetization and magnetoresistance of common alloy wires used in cryogenic instrumentation. Rev. Sci. Instrum..

[53] Swenson C., Markiewicz W. (1999). Persistent joint development for high field NMR. IEEE Trans. Appl. Supercond..

[54] Cheng J., Liu J., Ni Z., Cui C., Chen S., Song S., Li L., Dai Y., Wang Q. (2012). Fabrication of NbTi Superconducting Joints for 400-MHz NMR Application. IEEE Trans. Appl. Supercond..

[55] Clarke J., Braginski A.I. (2004). The SQUID Handbook: Fundamentals and Technology of SQUIDs and SQUID Systems.

[56] Halbritter J. (1987). On the oxidation and on the superconductivity of niobium. Appl. Phys. A.

[57] Mizumaki S., Yamamoto A. (1997). Experimental study of current sharing and transfer in superconductor joint. IEEE Trans. Appl. Supercond..

[58] Karvonen E., Rayroux J.M. (1970). Electrical Connection Between Superconductors.

[59] Nuding J.M. (1969). Method of Making a Superconductive Joint.

[60] Cheng J., Li L., Zhou F., Liu J., Cui C., Hu X., Dai Y., Yan L., Cheng S., Li Y. (2015). Contact Resistance Properties of Cold-Pressing Superconducting Joints. IEEE Trans. Appl. Supercond..

[61] Thornton R.F. (1986). Superconducting Joint for Superconducting Wires and Coils.

[62] Patel D., Kim S.H., Qiu W., Maeda M., Matsumoto A., Nishijima G., Kumakura H., Choi S., Kim J.H. (2019). Niobium-titanium (Nb-Ti) superconducting joints for persistent-mode operation. Sci. Rep..

[63] Faley M.I., Kostyurina E.A., Kalashnikov K.V., Maslennikov Y.V., Koshelets V.P., Dunin-Borkowski R.E. (2017). Superconducting Quantum Interferometers for Nondestructive Evaluation. Sensors.

[64] Ohtani R., Hayashi K., Sagawa M., Ariyoshi S., Tanaka S. (2021). Estimation of Critical Current of HTS RF-SQUID. J. Phys. Conf. Ser..

[65] Leupold M.J., Iwasa Y. (1976). Superconducting joint between multifilamentary wires 1. Joint-making and joint results. Cryogenics.

[66] Morano M.J. (2023). Development of Hardware and Simulations for the nEDM@SNS Experiment. Ph.D. Thesis.

[67] Brittles G. (2016). Persistent Current Joints Between NbTi Superconducting Wires. Ph.D. Thesis.

